# Green‐Synthesized Silver Nanoparticles Against *Acinetobacter baumannii*: A Systematic Review of Antibacterial Activity, Mechanisms of Action, Toxicity, and Antibiofilm Effects

**DOI:** 10.1002/mbo3.70378

**Published:** 2026-08-19

**Authors:** Shahla Shahbazi, Kimia Rafieibuzhani, Keyghobad Ghadiri, Yadollah Bahrami, Roya Chegene Lorestani, Maryam Meskini, Samira Sabzi, Mosayeb Rostamian

**Affiliations:** ^1^ Infectious Diseases Research Center Health Policy and Promotion Institute, Kermanshah University of Medical Sciences Kermanshah Iran; ^2^ Department of Comparative Biomedicine and Food Science Master's Program in Biotechnologies for Food Science, University of Padua Padua Italy; ^3^ Department of Medical Biotechnology Faculty of Medicine, Kermanshah University of Medical Sciences Kermanshah Iran; ^4^ Department of Medical Biotechnology School of Medicine, College of Medicine and Public Health, Flinders University Adelaide Australia; ^5^ Medical Biology Research Center, Health Technology Institute Kermanshah University of Medical Sciences Kermanshah Iran; ^6^ Student Research Committee Kermanshah University of Medical Sciences Kermanshah Iran; ^7^ Department of Mycobacteriology and Pulmonary Research Pasteur Institute of Iran (IPI) Tehran Iran; ^8^ Microbiology Research Center, Pasteur Institute of Iran Tehran Iran; ^9^ Medical Microbiology Research Center Qazvin University of Medical Sciences Qazvin Iran; ^10^ Department of Microbiology and Immunology School of Medicine, Qazvin University of Medical Sciences Qazvin Iran

**Keywords:** *Acinetobacter baumannii*, antibacterial activity, biofilm inhibition, multidrug resistance, silver nanoparticles

## Abstract

*Acinetobacter baumannii* is a major multidrug‐resistant nosocomial pathogen, and new antimicrobial strategies are urgently needed. Green‐synthesized silver nanoparticles (AgNPs) have emerged as promising candidates because of their broad antibacterial activity and potentially improved biocompatibility. This systematic review evaluated the antibacterial effects of pure, plant‐mediated AgNPs against *A. baumannii* isolates. PubMed, Scopus, and Web of Science were searched from database inception to October 5, 2025 according to PRISMA guidelines. Eligible studies were original English‐language reports assessing pure green‐synthesized AgNPs against *A. baumannii*. Sixty‐three studies met the inclusion criteria. Green AgNPs were generally active against *A. baumannii* and more often showed stronger effects than the corresponding plant extracts. The most commonly proposed mechanisms were reactive oxygen species generation, membrane and cell–wall disruption, increased permeability, and damage to proteins, lipids, or DNA. Biofilm data were limited but suggested promising inhibitory potential. Toxicity findings were mixed, ranging from good biocompatibility to cytotoxicity in sensitive cell models. Plant‐mediated AgNPs show credible antibacterial potential against *A. baumannii*, but standardized synthesis, rigorous characterization, biofilm‐focused testing, and in vivo safety studies are still needed before clinical translation.

## Graphical abstract

Schematic overview of the main antibacterial mechanisms proposed for green‐synthesized silver nanoparticles (AgNPs) against *Acinetobacter baumannii*. The figure summarizes the phytosynthetic pathway used to produce AgNPs (right panel) and the major reported antibacterial effects, including reactive oxygen species generation, oxidative stress, membrane and cell–wall damage, increased permeability, lipid peroxidation, protein denaturation, DNA damage, inhibition of replication/translation, efflux pump inhibition, porin suppression, respiratory‐chain interference, apoptosis‐related effects, and antibiofilm activity. Overall, the diagram highlights the multifactorial nature of AgNP action and the contribution of plant‐derived phytochemicals as reducing and stabilizing agents during nanoparticle formation. The frequency of reporting is provided to reflect how often each mechanism was described in the included studies and should not be interpreted as a formal measure of the quality or certainty of evidence.

## 1 Introduction

Antimicrobial resistance represents one of the most pressing global health threats of the 21st century. Bacterial pathogens that have developed resistance to multiple classes of antibiotics severely limit treatment options and contribute to increased morbidity, mortality, and healthcare costs worldwide. Among these, *Acinetobacter baumannii* stands out as a particularly formidable opportunistic pathogen. This Gram‐negative coccobacillus thrives in hospital environments, especially intensive care units, where it causes severe infections, such as ventilator‐associated pneumonia, bloodstream infections, and wound infections (Ibrahim et al. 2021). Its remarkable ability to survive on dry surfaces for extended periods and its propensity to acquire diverse resistance mechanisms have made it a major concern in healthcare settings (Chegene Lorestani et al. 2023; Mea et al. 2021; Nguyen and Joshi 2021).

Multidrug‐resistant (MDR) and extensively drug‐resistant (XDR) strains of *A. baumannii* have become increasingly prevalent (Khoshnood et al. 2023; Zhang et al. 2024). These strains are often carbapenem‐resistant *A. baumannii* (CRAB) or exhibit resistance to other last‐resort antibiotics, leading to high treatment failure rates in critically ill patients (Kubin et al. 2025; Moghadam et al. 2025; Zhang et al. 2024). The pathogen's success stems from multiple resistance mechanisms, including enzymatic degradation of antibiotics, efflux pumps, altered membrane permeability, and biofilm formation, which collectively shield it from both drugs and host immune responses. Conventional antibiotic stewardship and infection control measures have proven insufficient to curb its spread, highlighting the urgent need for alternative therapeutic or preventive strategies that can bypass these resistance pathways (Bahrami et al. 2025; Heidarinia et al. 2025; Iovleva et al. 2025; Meskini et al. 2022).

In this context, silver nanoparticles (AgNPs) have emerged as promising candidates for combating MDR bacteria (Selvam et al. 2026). Silver has a long history of antimicrobial use, but its nanoscale form offers distinct advantages due to its high surface area‐to‐volume ratio, which enhances interaction with bacterial cells (Rodrigues et al. 2024). AgNPs can disrupt cell membranes, generate reactive oxygen species (ROS), interfere with protein function, and inhibit biofilm formation through multiple simultaneous mechanisms (Rodrigues et al. 2024). This multitarget action may reduce the likelihood of resistance development compared with traditional single‐target antibiotics. However, the synthesis method significantly influences the physicochemical properties, biocompatibility, and environmental impact of the resulting nanoparticles.

Traditional chemical and physical methods for producing AgNPs often rely on toxic reducing agents, organic solvents, and high‐energy inputs, which can leave harmful residues and raise concerns about cytotoxicity and ecological safety (Jiang et al. 2025). In contrast, green synthesis approaches utilizing plant extracts offer a more sustainable and potentially biocompatible alternative. Plant‐derived phytochemicals such as polyphenols, flavonoids, and terpenoids serve dual roles as reducing and capping agents, enabling the production of stable AgNPs under mild conditions without hazardous chemicals. These green‐synthesized AgNPs frequently demonstrate enhanced antibacterial efficacy, lower toxicity to mammalian cells, and improved environmental compatibility (Zehra et al. 2025). The diversity of plant species also enables tailoring nanoparticle properties to specific bioactive compounds, potentially leading to more effective formulations against challenging pathogens, such as *A. baumannii*.

Despite growing interest in green‐synthesized AgNPs, the existing literature on their specific activity against *A. baumannii* remains fragmented. Individual studies vary widely in plant sources, synthesis protocols, nanoparticle characterization, bacterial strains, and antimicrobial assay methodologies. This heterogeneity makes it difficult to draw robust conclusions about overall effectiveness, optimal conditions, or potential clinical translation. While several narrative reviews have addressed green AgNPs broadly (F. Arshad et al. 2024; Dubey et al. 2023; Huq et al. 2022; Zehra et al. 2025), a focused systematic evaluation of their performance specifically against *A. baumannii* is still needed.

The present study aims to systematically review the available evidence on the antibacterial effects of pure, green‐synthesized AgNPs against *A. baumannii* isolates. This systematic review seeks to clarify the potential of plant‐mediated AgNPs as an alternative strategy against this critical MDR pathogen and to identify directions for future optimization and translational research.

## 2 Methods

### 2.1 Study Design and Search Strategy

This study was conducted as a systematic review in accordance with the updated Preferred Reporting Items for Systematic Reviews and Meta‐Analyses (PRISMA) guidelines (Page et al. 2021) (Table S1). The search period extended from database inception to October 5, 2025. Relevant studies were retrieved from three major databases: Web of Science, PubMed, and Scopus. The search terms included “nanoparticle,” “nanomaterial,” “silver,” “AgNP,” “nanosilver,” “metallic nanoparticle,” “metal‐based nanoparticles,” “nanometals,” “nanostructured silver,” “silver colloids,” “silver nanocomposite,” “green synthesis,” “biosynthesis,” “phytosynthesis,” “plant‐mediated synthesis,” “plant,” “phytochemical,” “antibacterial,” “antimicrob,” “antimicrobial,” “bactericidal,” “bactericidal,” “microbicide,” “microbicidal,” “inhibition,” “inhibitor,” “*Acinetobacter baumannii*,” “*A. baumannii*,” “Gram‐negative coccobacillus,” and “MDRAB.” The keywords were used individually or in combination using appropriate “AND” and/or “OR” Boolean operators.

Table S1. PRISMA 2020 checklist showing the location of reported items in the manuscript.

### 2.2 Eligibility Criteria

Original research articles that were relevant to evaluating green‐synthesized AgNPs against *A. baumannii* isolates and were published in English up to October 5, 2025 were included.

The exclusion criteria were as follows: reviews, books, book chapters, letters to the editor, commentaries, editorials, news items, case reports, conference abstracts, protocols, and non‐English publications. In addition, the following studies were excluded: those using nonplant sources (such as fungi and algae); in silico studies; animal or veterinary studies; studies that reported natural sources but did not identify the plant source; studies on plant‐derived compounds without specifying the plant origin; studies on bacteria other than *A. baumannii*; studies involving nanoparticles other than AgNPs; studies using combined metals (e.g., bimetallic Ag‐Cu nanoparticles); studies involving nanocomposites, conjugated or decorated (nonpure) AgNPs, or AgNPs mixed with other molecules; studies with insufficient or improper data; studies that were unable to culture *A. baumannii*; studies reporting mixed Gram‐negative bacteria without separately presenting data for *A. baumannii*; and studies for which the full text was unavailable. Notably, studies whose main objective was not the use of pure AgNPs but also reported data on pure AgNPs were excluded, as they could introduce bias and potentially overestimate the effects of pure AgNPs.

### 2.3 Study Selection

All records retrieved from the selected databases were imported into reference management software, where duplicate entries were identified and removed. Two reviewers independently screened the remaining studies by examining their titles and abstracts against the predefined eligibility criteria. The full texts of the potentially relevant articles were then assessed in detail, and data were extracted using a standardized, predesigned form. Any disagreement between the reviewers was resolved through discussion and consultation.

### 2.4 Extracted Data

From the final eligible studies, the following information was extracted: first author, publication year, country, scientific name and family of the plant, plant material used (fresh or dried), plant part employed for synthesis, extraction solvent, extraction method, reaction conditions, synthesis approach, AgNP size, AgNP shape, zeta potential/stability, crystallinity/phase, *A. baumannii* strain, strain source, type of antibacterial assay, nanoparticle concentration, antibacterial findings, proposed mechanisms of action, and toxicity.

A meta‐analysis was not conducted because the included studies were highly heterogeneous in plant source, extraction procedures, comparator groups, bacterial strains, and antibacterial assay formats, and the outcomes were reported inconsistently.

### 2.5 Risk of Bias Assessment

The methodological quality of the included in vitro studies was evaluated using the Risk Of Bias In Nonrandomized Studies of Interventions (ROBINS‐I) tool (Sterne et al. 2016). Although this instrument was originally designed for nonrandomized clinical research, it was adapted here as a structured framework for assessing laboratory‐based experimental studies. Each article was independently appraised across seven domains: confounding, participant selection, intervention classification, deviations from the intended intervention, missing outcome data, outcome measurement, and selective reporting of results. On the basis of these domains, the overall risk of bias for each study was classified as low, moderate, serious, or critical.

## 3 Results

### 3.1 Search Findings

**Figure 1:**
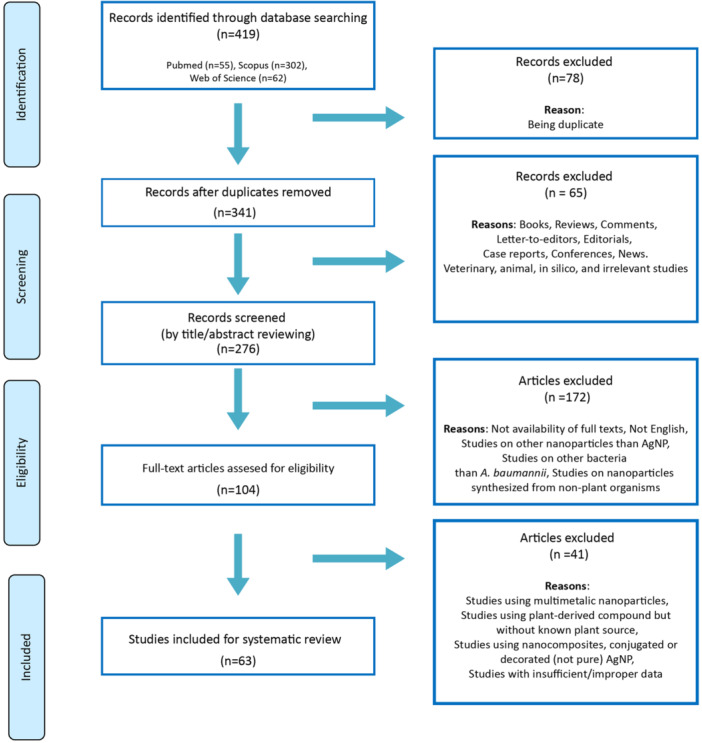
PRISMA flow diagram of the study selection process. AgNP, silver nanoparticle; PRISMA, Preferred Reporting Items for Systematic Reviews and Meta‐Analyses.

After the initial search, a total of 419 records were identified, including 55 from PubMed, 302 from Scopus, and 62 from Web of Science. After removing 78 duplicate records, 341 articles remained for screening. Title and abstract screening led to the exclusion of 237 records, including 65 in the first stage and 172 during the rescreening, leaving 104 articles for full‐text evaluation. Of these, 41 did not meet the eligibility criteria and were excluded, resulting in 63 studies included in the systematic review (Figure 1).

All 63 included studies were assessed using the adapted ROBINS‐I tool. Overall, the studies generally demonstrated a low risk of bias in domains related to participant selection and intervention classification. In contrast, moderate concerns were more commonly observed in domains, such as confounding and outcome measurement, which are especially difficult to control in in vitro research. No study was judged to have an overall low risk of bias, and all included studies were ultimately classified as having a moderate overall risk of bias. A detailed summary of the risk‐of‐bias assessment across all domains is presented in Table S2.

### 3.2 General Data

The first study was published in 2012, and the number of publications increased thereafter. The highest number of studies was published in 2024, with 14 records. Most reports originated from Iran (14), India (9), and Saudi Arabia (8), followed by Thailand (5), Turkey (5), Iraq (3), and Pakistan (3). Two studies were reported from China, Egypt, Morocco, Indonesia, and Taiwan, while one study each was reported from Brazil, Ethiopia, Libya, South Africa, and South Korea. In addition, one study was conducted across two countries, Saudi Arabia and India (Table 1A).

**Table 1A:** Studies characteristics—Part I.

Study ID	First author	Published year	Country	Plant	Plant family	Plant form	Used component for the synthesis	Extraction solvent	Extract method	Reaction conditions	Synthesis method	Reference
S1	Abdellatif	2022	Saudi Arabia	*Thymus vulgaris*	Lamiaceae	Dried	Leaves	Distilled water	Maceration	Magnetic stirring for 24 h	Green‐chemical	Abdellatif et al. (2022)
				*Mentha piperita*	Lamiaceae		Leaves					
				*Zingiber officinale*	Zingiberaceae		Roots					
S2	Abdussalam‐Mohammed	2025	Libya	*Hyoscyamus muticus*	Solanaceae	Dried	Leaves	Water to ethanol mixture (7:3)	Maceration	RT, pH: 9.17, 4 h	Green‐chemical	Abdussalam‐Mohammed et al. (2025)
S3	Abootalebi	2021	Iran	*Ferula asafoetida*	Apiaceae	Dried	Leaves and other aerial parts	Deionized water	NA	Green: 25°C, 48 h, shaking; chemical: ice‐cold temperature, 24 h, dark environment.	Green‐chemical	Abootalebi et al. (2021)
S4	Ajlouni	2022	Saudi Arabia	*Anthemis pseudocotula*	Asteraceae	Fresh	Aerial parts	Water	Reflux extraction	~3 h, 80°C	Thermal green	Ajlouni et al. (2022)
S5	Akar	2023	Turkey	*Equisetum arvense*	Equisetaceae	Dried	Leaves	Water	Soxhlet extraction	RT, 4 h, dropwise addition under stirring	Green	Akar et al. (2024)
S6	Akay	2024	Turkey	*Rumex* sp.	Polygonaceae	Dried	Leaves	Water	Soxhlet extraction	RT, 4 h, dropwise addition under stirring	Green	Akay et al. (2024)
S7	Akther	2019	India	*Terminalia arjuna*	Combretaceae	Dried	Barks	Methanol and water	NA	90°C, frequency: 2.45 GHz, agitation: constant stirring	Microwave‐assisted green	Akther et al. (2019)
S8	Alhamdi	2024	Egypt	*Lens culinaris*	Fabaceae	Dried	Seeds	70% Ethanol	Maceration	Ambient (overnight incubation in a dark place)	Green	Alhamdi et al. (2024)
S9	Alharbi‐1	2020	Saudi Arabia	*Neurada procumbens*	Neuradaceae	Fresh	Leaves	Water	Decoction	Optimized at five levels: RT, 35°C, 50°C, 75°C, 95°C	Thermal green	F. A. Alharbi and Alarfaj (2020)
S10	Alharbi‐2	2023	Saudi Arabia	*Senna alexandrina*	Fabaceae	Dried	Leaves	Deionized water	Decoction	220 rpm, 24 ± 1°C, 10 min	Sunlight‐assisted green	N. S. Alharbi et al. (2023)
S11	Almudhafar	2022	Iraq	*Eragrostis tef*	Poaceae	Dried	Seeds	Deionised water	Decocation	RT for 4 h	Green‐chemical	Almudhafar and Al‐Hamdani (2022)
				*Vitellaria paradoxa*	Sapotaceae							
S12	Al‐Otibi	2023	Saudi Arabia	*Camellia sinensis*	Theaceae	Dried	Leaves	Distilled water	Decoction	Shaking the incubator under dark conditions for 24 h at 25°C	Green	Al‐Otibi et al. (2023)
S13	AlSalhi‐1	2016	Saudi Arabia	*Pimpinella anisum*	Apiaceae	Dried	Seeds	Water	Maceration	RT, 96 h	Green	AlSalhi et al. (2016)
S14	AlSalhi‐2	2019	India	*Memecylon umbellatum*	Melastomataceae	NA	Leaves	Methanol	Column chromatography	RT, few hours	Green‐chemical	AlSalhi et al. (2019)
S15	Alsharari	2024	Saudi Arabia	*Rosa* sp.	Rosaceae	Dried	Flowers petal	Deionized water	Decoction	Irradiation: 5 cycles of 150 s each (850 W) with 2‐min intervals	Microwave‐assisted green	Alsharari et al. (2024)
S16	AL‐Zubaidi	2025	Iraq	*Zamioculcas zamiifolia*	Araceae	NA	Leaves	Water	Decoction	Initial heating: 70°C for 30 min, incubation: 24 h in the dark	Thermal green	AL‐Zubaidi and Kamil (2025)
S17	Aman	2023	India	*Citrus pseudolimon*	Rutaceae	NA	Fruit peel	20% Methanol	Maceration	Temperature: 70°C (reaction) and 60°C (drying), pH: 9, Consistent stirring	Thermal green	Aman et al. (2023)
S18	Arshad	2021	Pakistan	*Aloe vera*	Asphodelaceae	Fresh	Leaves	Distilled water	Decoction	4–6 h at 65°C	Thermal green	H. Arshad et al. (2022)
S19	Baghayeri	2017	Iran	*Salvia leriifolia*	Lamiaceae	Dried	Leaves	Distilled water	Decoction	30°C (using a shaking stirrer), 48 h	Thermal green	Baghayeri et al. (2018)
S20	Barabadi	2024	Iran	*Punica granatum*	Lythraceae	Dried	Flowers	Distilled water	Maceration	28°C (incubation) and 40°C (final drying), pH range: optimized across seven levels (4, 5, 6, 7, 8, 9, and 10), Time: 48 h for synthesis	Green	Barabadi et al. (2024)
S21	Behdad	2020	Iran	*Acroptilon repens*	Asteraceae	NA	Aerial parts	Ethanol	Maceration	RT for reaction; 40°C for 2 h (drying). Time: 5 min of stirring, then undisturbed until color change (dark brown)	Green	Behdad et al. (2020)
S22	Buapoon	2021	Thailand	*Clitoria ternatea*	Fabaceae	Fresh	Flowers petal	Distilled water	NA	50°C for 15 min, pH: 10	Alkaline green	Buapoon et al. (2021)
S23	Calhan	2020	Turkey	*Onosma sericea*	Boraginaceae	Dried	Roots	Distilled water	Maceration	pH 8, 25°C	Green	DOğan Çalhan and Gündoğan (2020)
S24	Ceylan	2024	Turkey	*Olea europaea*	Oleaceae	Dried	Leaves	Distilled water	Decoction	25°C (reaction) and 65°C (oven drying for 48 h)	Green	Ceylan and Hacıoğlu Doğru (2025)
S25	Choi	2021	South Korea	*Areca catechu*	Arecaceae	NA	NA	Distilled water	Hydrothermal	RT for 4 h in the dark	Green	Choi et al. (2021)
S26	Deonas	2024	Brazil	*Anadenanthera colubrina*	Fabaceae	NA	NA	Water	NA	24 h	Green	Deonas et al. (2024)
S27	Devanesan	2021	Saudi Arabia/India	*Carica papaya*	Caricaceae	Dried	Leaves	Methanol	Soxhlet extraction	NA	Green‐chemical	Devanesan et al. (2021)
S28	Ebrahimzadeh	2019	Iran	*Crataegus pentagyna*	Rosaceae	Fresh	Fruits	Methanol	Maceration	80°C, pH 12 for 2 h under vigorous stirring	Green	Ebrahimzadeh et al. (2020)
S29	Eroglu	2025	Turkey	*Onosma mutabilis*	Boraginaceae	Dried	Roots	Distilled water	Maceration	Both RT and 85°C; acidic (pH 2) and basic (pH 8) conditions	Green	Eroğlu and Valiyeva (2025)
S30	Gautam	2023	India	*Ocimum tenuiflorum* (syn. *Ocimum sanctum*)	Lamiaceae	Fresh	Leaves	Ultrapure water	Decoction	Constant stirring at 400 rpm during the 30‐min dropwise addition. RT in a dark room for 1 h	Green	Gautam et al. (2023)
S31	Geyesa	2024	Ethiopia	*Verbascum sinaiticum*	Scrophulariaceae	Dried, powder	Leaves	Methanol	Maceration	NA	Green	Geyesa et al. (2024)
S32	Ghosh	2012	India	*Dioscorea bulbifera*	Dioscoreaceae	Dried	Tuber	Water	Decoction	4°C–50°C with reflux	Thermal green	Ghosh et al. (2012)
S33	Hashemi‐1	2021	Iran	*Ferula persica*	Apiaceae	NA	Aerial parts	NA	Maceration	65°C, pH: 12, 60 min vigorous stirring	Alkaline green	Hashemi et al. (2021)
S34	Hashemi‐2	2022	Iran	*Berberis vulgaris*	Berberidaceae	Dried	Fruits	Metahnol	Maceration	Stirring at 85°C, pH 10	Thermal green	Hashemi et al. (2022)
S35	Hashemi‐3	2023	Iran	*Mentha pulegium*	Lamiaceae	Dried	Aerial parts	Metahnol	Maceration	65°C, 30 min	Thermal green	Hashemi et al. (2023)
				*Crocus caspius*	Iridaceae							
S36	Hou	2023	China	*Perilla frutescens*	Lamiaceae	Dried	Leaves	Methanol	Maceration	55°C–60°C, 1 h heating; 30 min cooling in the dark	Thermal green	Hou et al. (2023)
S37	Hudaya‐1	2024	Indonesia	*Artocarpus heterophyllus*	Moraceae	Dried	Leaves	Deionized water	Decoction	90°C, 30 min	Microwave‐assisted green	Hudaya, Situmorang, et al. (2024)
S38	Hudaya‐2	2024	Indonesia	*Toona sureni*	Meliaceae	Dried	Leaves	96% Ethanol	NA	37°C, 24 h, 150 rpm in the dark	Green	Hudaya, Mukti, et al. (2024)
S39	Khatami	2018	Iran	*Cichorium intybus*	Asteraceae	NA	Seeds	Deionized water	Maceration	25°C, pH 3	Green‐chemical	Khatami et al. (2018)
S40	Khojasteh‐Taheri	2023	Iran	*Salvadora persica*	Salvadoraceae	NA	NA	Distilled water	Maceration	1000 rpm at RT, 48 h	Green‐chemical	Khojasteh‐Taheri et al. (2023)
				*Caccinia macranthera*	Boraginaceae							
S41	Kumar	2025	India	*Valeriana wallichii*	Caprifoliaceae	Dried	Roots	Methanol	Soxhlet extraction	Incubated in a dark room at RT	Green‐chemical	Kumar et al. (2025)
S42	Kumkoon	2023	Thailand	*Morus alba*	Moraceae	Fresh	Leaves	Sterile water	Maceration	75°C, 2 h	Green‐chemical	Kumkoon et al. (2023)
S43	Lediga	2018	South Africa	20 plants, 2 tested on *Acinetobacter baumannii*: *Eucomis autumnalis* and *Sclerocarya birrea*	Hyacinthaceae (*E. autumnalis*), Anacardiaceae (*S. birrea*)	Dreid	Leaves, roots, or barks	Deionized water	Decoction	80°C, 10 min with stirring	Thermal green	Lediga et al. (2018)
S44	Megdar‐1	2024	Morocco	*Lavandula mairei*	Lamiaceae	Dried	Aerial parts	Distilled water	Decoction	37°C for 24 h with continuous stirring	Thermal green	El Megdar et al. (2024)
S45	Megdar‐2	2025	Morocco	*L. mairei*	Lamiaceae	Dried	NA	Distilled water	Decoction	Centrifugation at 10,000 rpm for 30 min	Thermal green	El Megdar et al. (2025)
S46	Mickymaray	2019	Saudi Arabia	*Sisymbrium irio*	Brassicaceae	Fresh	Leaves	Ultrapure Milli Q water	Decoction	Stirred at 35°C	Thermal green	Mickymaray (2019)
S47	Mishra	2018	India	*Anogeissus acuminata*	Combretaceae	NA	Leaves	Water	NA	Standing at RT for 24 h	Green‐chemical	Mishra and Padhy (2018)
S48	Moorthy‐1	2021	Taiwan	*Momordica charantia*	Cucurbitaceae	Dried	Fruits	80% Ethanol	Soxhlet extraction/decoction	Vigorous stirring and refluxed at 100°C for 2 h	Thermal green	Moorthy et al. (2021)
S49	Moorthy‐2	2022	Taiwan	*Imperata cylindrica*	Poaceae	Dried	Leaves	Water	NA	15 min	Thermal green	Moorthy et al. (2022)
S50	Moradi	2024	Iran	*Thymus daenensis*	Lamiaceae	Dried	Flowering branches	Deionized water	Decoction	Shaker at ambient temperature for 30 min	Green‐chemical	Moradi et al. (2025)
S51	Naz	2017	Pakistan	*Azadirachta indica*	Meliaceae	NA	Seeds	Distilled water	Maceration	Mixing at pH 8, for 30 min	Green‐chemical	Naz et al. (2017)
S52	Ni	2024	China	*Ginkgo biloba*	Ginkgoaceae	Dried	Leaves	Deionized water	Decoction	pH range: 3, 5, 7, 9, and 11; temperature range: 30°C−90°C. Time range: 30, 45, 60, 75, and 90 min	Green‐chemical	Ni et al. (2024)
S53	Nithin	2022	India	*Mimusops elengi*	Sapotaceae	Dried	Flowers	Water	Maceration	Incubation in the dark	Green‐chemical	Charlz Nithin et al. (2022)
S54	Ontong	2019	Thailand	*Senna alata*	Fabaceae	Dried	Barks	Distilled water	Maceration	On a rotary shaker (150 rpm) at 60°C for 3, 6, 9, and 12 h under dark conditions	Thermal green	Ontong et al. (2019)
S55	Paosen	2019	Thailand	*Eucalyptus citriodora*	Myrtaceae	NA	Leaves	Ethanol	NA	28°C for 16 h on rotator shaker at 150 rpm in the dark	Green‐chemical	Paosen et al. (2019)
S56	Ramya	2020	India	*Z. officinale*	Zingiberaceae	Fresh	Roots	Distilled water	Decocation	NA	Green‐chemical	Ramya et al. (2020)
S57	Said	2024	Egypt	*Lawsonia inermis*	Lythraceae	Dried	Leaves	Distilled water	Maceration	Vigorous stirring	Green‐chemical	Said et al. (2024)
S58	Saleh	2021	Iraq	*Cinnamomum zeylanicum*	Lauraceae	Dried	Barks	Distilled water	Decocation	Incubated at RT with continuous stirring for 18 h	Green‐chemical	Reyam Ayad (2021)
S59	Salehi	2016	Iran	*Artemisia marschalliana*	Asteraceae	Dried	Aerial parts	Deionized water and ethanol (1:1)	NA	Continuously stirred for 5 min at RT	Green‐chemical	Salehi et al. (2016)
S60	Shamsoddini	2024	Iran	*A. repens*	Asteraceae	Dried	NA	80% Ethanol	Maceration	Continuous stirring at RT for 1 h	Green‐chemical	Shamsoddini et al. (2025)
S61	Shirzadi‐Ahodashti	2021	Iran	*Convolvulus fruticosus*	Convolvulaceae	Dreid dried	Aerial parts	Methanol	Percolation	Various temperatures (RT, 45°C, 60°C, and 85°C), pH (6, 8, 10, and 12), time (1, 2, 3, 4, 5, and 24 h), and silver nitrate concentration (1–10 mM)	Thermal green	Shirzadi‐Ahodashti et al. (2021)
S62	Wintachai	2018	Thailand	*E. citriodora*	Myrtaceae	NA	Leaves	95% Ethanol	NA	28°C, shaking at 150 rpm, dark, 16 h	Green‐chemical	Wintachai et al. (2019)
S63	Younas	2023	Pakistan	*Moringa oleifera*	Moringaceae	Fresh	Leaves	Distilled water	Decoction	Ambient temperature	Green‐chemical	Younas et al. (2023)

Abbreviations: AFM, atomic force microscopy; AgNO_3_, silver nitrate; AgNP, silver nanoparticle; AWD, agar well diffusion; BMD, broth microdilution; CFU, colony‐forming units; DD, disk diffusion; DLS, dynamic light scattering; FCC, face‐centered cubic; HTEC, human tracheal epithelial cells; IZ, inhibition zone; MBC, minimum bactericidal concentration; MDR, multidrug resistant; MIC, minimum inhibitory concentration; MRAB, Methicillin resistant *A. baumannii*; NA, not available; OD, optical density; REMA, resazurin microtiter assay; RT, room temperature; SEM, scanning electron microscope; TEM, transmission electron microscopy; XDR, extensive drug‐resistant; XRD, X‐ray powder diffraction.

### 3.3 Plants and Extraction

Sixty‐six different plant species were used, and most of them were unique to individual studies. The only plants reported in more than one study were *Eucalyptus citriodora* (two studies), *Lavandula mairei* (two studies), and *Zingiber officinale* (two studies). The most frequently reported plant families were Lamiaceae (eight records), Asteraceae (five records), and Fabaceae (five records) (Table 1A).

Most plants were used in dried form (40 reports), whereas fresh plants were used in 10 studies; the plant form was not reported in 13 studies. The plant component used for synthesis varied according to the species and included leaves (29 reports), aerial parts (eight reports), roots (six reports), flowers (two reports), seeds (two reports), bark (four reports), fruits (four reports), and tubers (one report); this information was not available in nine studies.

The most commonly used extraction methods were maceration (24 records) and decoction (22 records), followed by Soxhlet extraction (six records). Single reports described the use of column chromatography, hydrothermal extraction, percolation, and reflux extraction. Water (46 records), methanol (11 records), and ethanol (nine records) were the most frequently used extraction solvents (Table 1A).

Reaction conditions also varied widely across studies, depending on factors such as the extraction method and solvent used. These details are presented in Table 1A.

### 3.4 AgNP Synthesis and Characterization

As expected, all included studies used green synthesis approaches. Of these, 16 studies described the method simply as green synthesis, 23 used green chemistry‐based synthesis, 18 used thermal green synthesis, three used microwave‐assisted green synthesis, two used alkaline green synthesis, and one employed sunlight‐assisted green synthesis for nanoparticle production (Table 1A).

The size of the AgNPs varied across studies, although most particles were smaller than 100 nm (64 reports). Among these, the majority (46 reports) were below 50 nm. One study reported particles larger than 100 nm, while another reported a wide size range across different analytical methods, from 12 to 70–700 nm, suggesting possible agglomeration. It should be noted that some studies reported more than one nanoparticle size, which is why the number of size reports differs from the number of included studies (Table 1B).

**Table 1B:** Studies characteristics—Part II.

Study ID	AgNP size (nm)	AgNP shape	Zeta potential/stability	Crystallinity/phase (from XRD)	*Acinetobacter baumannii* strain	Source of strain	Antibacterial test type	AgNP concentration (µg/mL)	Mechanistic Insights	Toxicity results
S1	*Thymus vulgaris*: 16.59 ± 0.78	Spherical	NA	NA	NA	Clinical	AWD, BMD	AWD: 50 μL; BMD: 2.26, 1.13, 0.56, 0.28, 0.14, 0.0716, and 0.04 mg/mL	Reactive oxygen species (ROS) generation; increased membrane permeability	NA
	*Mentha piperita*: 45.94 ± 1.07									
	*Zingiber officinale*: 81.04 ± 0.98									
S2	17.3 ± 4.5	Spherical	−64.42 mV high stability	NA	ATCC 17978	Clinical	DD	1, 2, 3, 4, and 6 µg/mL	ROS generation; Ag^+^ release; protein denaturation; DNA damage	NA
S3	~42	NA	NA	Hexagonal structure with silver in cubic form	Clinical	Clinical	AWD, BMD	BMD: 0.5–250 µg/mL, AWD: up to 200 µg/mL	ROS generation; membrane damage	MCF‐7 IC50 100 µg/mL
S4	~20	Spherical	NA	FCC	MDR	Clinical	BMD, Tissue culture plate assay (antibiofilm activity)	19–5000 µg/mL	ROS generation	NA
S5	40–60	NA	−107 mV	NA	Clinical	Clinical	BMD, air–liquid interface assay	BMD: 500–62.5 µg/mL; interface assay: 62.5 µg/mL	Membrane disruption; ROS generation; enzyme inactivation	A549 IC50 156 µg/mL
S6	< 100	Spherical	NA	NA	MDR	Clinical	AWD, BMD, quantitative biofilm formation assay	AWD: 500 µg/mL; biofilm: 125 µg/mL	Membrane neutralization; increased permeability; ROS generation; protein/nucleic acid interaction	NA
S7	20–100	Spherical	NA	NA	ATCC 19606	Reference strain	AWD	20, 30, and 50 µg/mL	ROS generation; membrane neutralization; increased permeability; resistance‐gene downregulation	NA
S8	158.3 ± 4.37	NA	−14.3 mV	FCC	XDR isolate M18	NA	AWD	NA	Apoptotic pathway activation	Cytotoxic against MCF‐7, HCT‐116, and HepG2
S9	20–50	Spherical	NA	FCC	MDR	Clinical	AWD	10, 25, 50, 75, and 100 µg/mL	ROS generation; membrane damage; protein, lipid, and DNA damage	NA
S10	4–20	Spherical	NA	Crystalline	Haemolyticus	Reference strain	BMD	1.3 mg/mL	Cell membrane targeting	MCF‐7 IC50 61.9 ± 3.8 µg/mL
S11	*Eragrostis tef*: 12.59–34.60	NA	NA	NA	ATCC 17904	Clinical and ecological isolates	AWD	150 µg/mL	Membrane damage; oxidative stress	A549 IC50 46 µg/mL; HTECs nontoxic up to 320 µg/mL
	*Vitellaria paradoxa*: 29.05–83.94									
S12	35.761 ± 3.18	NA	−14.1 mV	FCC	ATCC 43498	Reference strain	BMD, DD	200 µg/disk	ROS generation; membrane adhesion/disruption; DNA replication inhibition; ribosome disruption	NA
S13	3.2–16 (average 8.3)	Spherical	NA	Cubic structure	Strain 4436	Clinical	AWD	10, 20, 30, 40, and 50 µg/mL	ROS generation; membrane damage; enzyme inactivation; protein and DNA damage	NA
S14	7–23 (average 15)	Spherical	NA	Crystalline	ATCC 19606	Reference strain	AWD	10, 25, 50, and 100 µg/mL	Membrane permeability interference; protein inactivation; DNA replication interference	MCF‐7 IC50 42.19 mg/mL
S15	26	Spherical	NA	Crystalline	MDR	Clinical	BMD	BMD: 200–3200 µg/mL	Cell membrane binding/penetration	NA
S16	23–65	Spherical	NA	FCC	NA	NA	AWD	1.5, 3.5, and 5.5 mL	Cell–wall interaction; respiratory inhibition	NA
S17	~40	Spherical	−31 mV	Crystalline	MDR	Clinical	CFU count	5 mg/mL	ROS accumulation; DNA damage; membrane disruption	RBCs/PBMCs: 4.58% hemolysis
S18	12.2 (crystalline size from XRD)	Spherical, oval, and some irregular shapes	−15.4 mV	FCC	MDR	Clinical	AWD, BMD	250, 500, 750, and 1000 µg/mL	Cell–wall/membrane disruption; ROS generation	NA
S19	27	Spherical	NA	FCC	NA	Clinical	DD	100 mg/mL	Free‐radical generation; increased membrane permeability	NA
S20	40.50 (*Z*‐average diameter)	Spherical	Stable: −19.5 mV	FCC	ATCC 19606	Reference strain	AWD, BMD	AWD: 1000 μg/mL, BMD: 16 μg/mL	ROS generation; cell–wall/membrane disruption	NA
S21	38.89	Spherical	−12.55 mV	FCC	MDR	Clinical	BMD	3.125–200 μg/mL	Efflux pump inhibition	NA
S22	10–30	Spherical	NA	FCC	ATCC 19606, PW001	Clinical	BMD	0.16–1.27 µg/mL	Membrane destruction	MCF‐7 IC50 76.63–169.77 µg/mL
S23	1–10 (AgNP1), 10–30 (AgNP2), and 30–50 (AgNP3)	Spherical	AgNP1: −42.9 ± 3.67 mV; AgNP2: −41.8 ± 2.33 mV; and AgNP3: −33.9 ± 1.55 mV	FCC	ATCC 02026	Reference strain	REMA	500 µg/mL	ROS generation; membrane damage; DNA toxicity	MCF‐7 cytotoxicity
S24	51–56	Spherical	NA	NA	ATCC 19606	Reference strain	BMD, DD	2.5 µg/mL	NA	NA
S25	20–30	Round, oval, and pebble‐like	NA	NA	KCTC 2508 and MDR (CCARM 12005)	Reference strain	AWD, BMD	AWD: 11.25–360 µg/mL; BMD: 0.7–360 µg/mL	Cell membrane destruction	NA
S26	75.62 ± 0.38	Spherical	−29 mV	FCC	AB141	Clinical	BMD	2.11 μM	Cell–wall/membrane damage; electron transport inhibition; DNA damage	Human erythrocytes CC50 961 µM
S27	7–22	Spherical	NA	Mono‐crystalline, FCC	NA	Clinical	AWD	25, 50, 75, and 100 µg/mL	ROS generation; membrane disruption	Vero cells: low toxicity; HepG2, MCF‐7; and A549: cytotoxicity
S28	25–45	Spherical	–19.6 mV	Crystalline	ATCC 19606 and MDR	Clinical and reference	BMD	0.11–14 µg/mL	NA	NA
S29	36.20–64.14 (via SEM)	Cubic	NA	Crystalline	ATCC 02026	Reference strain	REMA	0.24–500 µg/mL	NA	NA
S30	55; range: 29–54.9 (TEM)	Almost spherical to oval or rod‐shaped	–27 mV	NA	MDR	Clinical	AWD, BMD	AWD: 200 µg/mL; BMD: 2–256 µg/mL	Cell–wall/membrane damage	A549: low toxicity up to 125 µg/mL
S31	2–40	Spherical	–28.1 mV	Crystalline	ATCC 19606	Reference strain	AWD, BMD	500, 1000, 1500, and 2000 µg/mL	NA	NA
S32	8–20 (TEM); ~ 13.54 (DLS); crystallite size ~ 10 (XRD)	Triangular, spherical, and ellipsoidal	NA	FCC	NA	Clinical/Nosocomial (MDR isolates)	DD	10–1000 µg/disk	Increased membrane permeability	NA
S33	15	Spherical	NA	Crystalline	ATCC 29606	Reference strain	BMD	7.8–250 µg/mL	NA	AGS IC50 11.07 µg/mL; MCF‐7 IC50 21.28 µg/mL
S34	Average: 50 (TEM); range 50–70 (FE‐SEM)	NA	−26.6 mV	FCC	ATCC 29606 and clinical MDR	Clinical and Reference	BMD	up to 500 µg/mL	ROS generation; membrane penetration	AGS IC50 3.16 µg/mL; MCF‐7 IC50 9.37 µg/mL
S35	MP‐AgNPs: 34.5; CC‐AgNPs: 47.2	Spherical	NA	Crystalline	NA	Clinical	BMD	0.5–200 µg/mL	Cell membrane penetration	Low toxicity to HFF cells
S36	20–70	Spherical	−30 mV	FCC	NA	Pathogenic	DD	25 µg/mL	ROS generation	COLO205 IC50 59.57 µg/mL; B16F10 IC50 69.33 µg/mL
S37	97.3	Spherical	NA	FCC	NA	Clinical	BMD	62.5–250 μg/mL	Cell membrane penetration; disruption of cellular structure and biofilm integrity	NA
S38	64.2	Spherical	NA	FCC	MDR	Clinical	BMD	Up to 500 μg/mL	ROS generation; protein denaturation; increased membrane permeability	NA
S39	8	Globular/spherical	NA	Crystalline	ATCC 19606	Reference strain	AWD, BMD	0.19–400 μg/mL	NA	NA
S40	CmNPs: 14.8; SpNPs: 39.4	Spherical	CmNPs: 1.4 mV; SpNPs: −3.4 mV	Crystalline	MDR	Reference strain and clinical	DD	0.97–1000 μg/mL	Cell membrane disruption	L929 nontoxic
S41	12 (AFM); range of 100–120 (DLS); and 70–700 nm (SEM, suggesting agglomeration)	Spherical	NA	FCC	MDR	Clinical	AWD	1–100 µg/mL	ROS generation; DNA fragmentation; membrane disruption	L929 LC50 368 µg/mL
S42	20–44.5	Spherical	−14.5 mV	FCC	ATCC 17978 and ATCC 19606	Reference strain	BMD	2–512 µg/mL	ROS generation; DNA fragmentation	MCF‐7 IC50 18 µg/mL
S43	10–45	Spherical	NA	Crystalline	ATCC 19606	Clinical	BMD	NA	Oxidative stress	Brine shrimp lethality assay: LC50 14.12 µg/mL
S44	40–70	Spherical	NA	Cubic structure	MDR	Clinical	BMD	78–5000 µg/mL	DNA/proteins fragmentation	NA
S45	30–60	Spherical	NA	FCC	MDR	Clinical	BMD, Antibiofilm assay	16–4096 µg/mL	DNA/proteins fragmentation	NA
S46	24–50	Spherical	NA	FCC	MDR	Clinical	AWD	BMD: 6.25–100 µg/mL	ROS generation; membrane disruption	NA
S47	< 100	Spherical	NA	Crystalline	MDR	Clinical	AWD	5, 10, and 15 µg/mL	Membrane disruption	NA
S48	A‐BG‐AgNPs: 16.4 ± 4.9; E‐BG‐AgNPs: 9.6 ± 3.7	Spherical	Aqueous: −33.0 ± 0.6 mV; ethanolic: −32.7 ± 0.5 mV	Crystalline	ATCC 17978 and clinical isolates, including colistin‐resistant (CR32X), and imipenem‐resistant (IMP32X) strains	Reference strain and clinical	BMD	1–64 µg/mL	ROS generation; cell membrane disruption	Biocompatible
S49	Extract + NP: Decoction: 19.7 ± 4.8; and blender: 20.1 ± 5.5	Spherical	Extract + NP: Decoction: −3.5 ± 3.0 mV; blender: −26.0 ± 6.7 mV	Crystalline	ATCC 17978 and clinical isolates, including colistin‐resistant (CR32X), and imipenem‐resistant (IMP32X) strains	Reference strain and clinical	BMD	1–64 µg/mL	ROS generation; cell membrane disruption	Biocompatible
S50	10–50 (average of 4.09 via XRD)	Rather cube‐shaped	NA	Crystalline	NA	Clinical	AWD	125 and 8000 µg/mL	Protein denaturation; proton motive force loss; ROS‐mediated cell damage	Biocompatible
S51	13 (5–30)	Spherical	NA	FCC	NA	Reference strain	DD	12 µg/mL	Membrane perforation; leakage; lysis; plasma membrane protein denaturation	Biocompatible
S52	~ 8–9	Spherical	NA	FCC	ATCC 17978	Reference strain	BMD, AWD	0.5, 1, 2, 4, 8, 16, 32, and 64 µg/mL	ROS generation; increased membrane permeability	Normal cell lines (L929, rBMSCs, hPDLCs): noncytotoxic below 16 µg/mL
S53	72.68 (SEM)	Predominant Spherical	−19.4 mV	FCC	MTCC 1926 and SN 15	Clinical and reference strain	AWD	0.63–20 µg/mL	ROS generation; DNA replication inhibition	Low toxicity to humans
S54	10–30	Spherical	−18.76 ± 1.21 mV	FCC	ATCC 19606	Reference strain	BMD	NA	Membrane damage	NA
S55	17.51 (ranging 10–60)	Spherical	Between –31.48 ± 2.56 and –13.37 ± 0.98 mV	NA	ATCC 19606 and MDR	Clinical and Reference strain	BMD, time‐kill assay	0.02–0.36 µg/mL	Membrane damage	Human red blood cells: nontoxic
S56	10–20	Spherical	NA	FCC	ATCC 17978	Reference strain	AWD	10, 20, and 30 µL	Oxidative stress	HCT116: significant cytotoxicity
S57	28–60	Spherical	NA	Crystalline	MDR	Clinical	AWD	6.5, 12.5, 25, 50, and 100 ppm	Membrane disruption; respiratory enzyme inhibition	NA
S58	7.55–24.4	Spherical	NA	FCC	MDR	Clinical	AWD	10%, 25%, 50%, 75, and 100%	Membrane protein damage; DNA replication inhibition	NA
S59	5–50	Spherical	−31 mV	FCC	ATCC 19606	Reference strain	DD	25, 50, 75, and 100 ppm	Membrane disruption	NA
S60	30	Spherical	NA	FCC	ATCC 19606, ATCC 17978	Clinical	BMD	1.56–450 μg/mL	Porin expression inhibition	NA
S61	45	Spherical	−23.7 mV	FCC	ATCC 19606 and clinical isolate AB‐2	Reference strain and Clinical	BMD	0.7 mg/mL	Cell–wall destruction; interaction with ‐SH groups; DNA replication inhibition	Safe for human therapeutics
S62	8–15	Spherical	NA	NA	ATCC 19606, MDR NPRCOE 160575	Clinical and reference strain	BMD	0.05–0.18 µg/mL	DNA condensation; ROS generation	A549: no significant cytotoxicity up to 0.72 µg/mL; CC50 5.72 µg/mL
S63	25.235 ± 0.694	Spherical	NA	FCC	ATCC 19606	Reference strain	AWD	1000, 2000, and 3000 µg/mL	ROS generation	NA

*Note:* The study IDs and abbreviations used in this table are the same as those presented in Table 1A.

Most studies reported spherical AgNPs (51 studies), while cubic particles were described in two studies and mixed morphologies in four studies. Particle shape was not reported in six studies (Table 1B).

Zeta potential, which reflects nanoparticle surface charge and stability, was reported in only 24 studies. Among these, 14 studies found values between −30 and −10 mV, indicating a moderately negative surface charge; eight studies reported values of ≤ − 30 mV, suggesting strongly negative charge; one study reported a neutral zeta potential ranging from −10 to +10 mV; and one study reported two zeta potential values, one anionic and one neutral, for AgNPs produced by two different synthesis methods (Table 1B).

Crystallinity was reported in 52 studies. Of these, 32 described the nanoparticles as having a face‐centered cubic (FCC) structure, 17 reported them as crystalline without further specification, and three studies identified a cubic structure (Table 1B).

### 3.5 Bacteria and Antibacterial Tests

Forty‐one studies used clinical isolates, 28 studies used reference strains of *A. baumannii*, and two studies did not report the bacterial source. Some investigations used both clinical and reference strains. Regarding the *A. baumannii* strains, 21 studies evaluated MDR isolates without specifying the strain number. The most commonly reported reference strains were ATCC 19606 (17 records) and ATCC 17978 (seven records). Other strains were reported only sporadically, and 10 studies did not specify the bacterial strain used (Table 1B).

The most frequently used antibacterial assays were broth microdilution (BMD) (37 records), agar well diffusion (AWD) (27 records), and disk diffusion (DD) (nine records). Biofilm assays were reported in four studies, colony‐forming unit (CFU) counting in one study, and resazurin microtiter assay (REMA) in two studies. In addition, one study each reported an air–liquid interface assay, a time‐kill assay, and an optical density (OD)‐based assessment (Table 1B).

Because the AgNP concentrations used for antimicrobial testing varied widely across studies, they could not be summarized meaningfully as a single value; however, the full details are provided in Table 1B and may be useful for future research.

### 3.6 Mechanisms of AgNP Effects on *A. baumannii*

**Figure 2:**
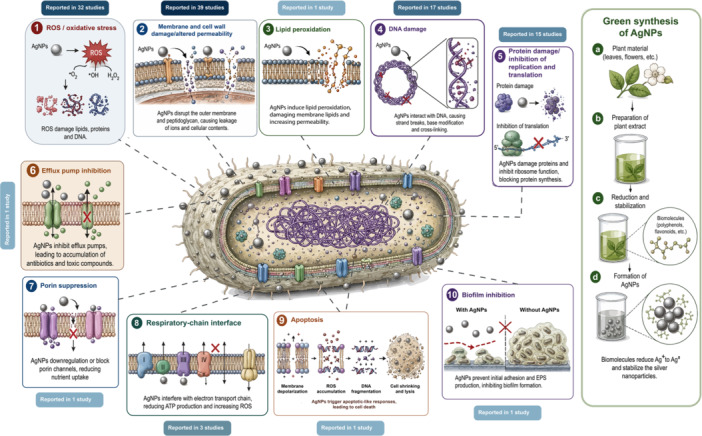
Schematic overview of the main antibacterial mechanisms proposed for green‐synthesized silver nanoparticles against *Acinetobacter baumannii*. The figure summarizes the phytosynthetic pathway used to produce silver nanoparticles (AgNPs) (right panel) and the major reported antibacterial effects, including reactive oxygen species (ROS) generation, oxidative stress, membrane and cell–wall damage, increased permeability, lipid peroxidation, protein denaturation, DNA damage, inhibition of replication/translation, efflux pump inhibition, porin suppression, respiratory‐chain interference, apoptosis‐related effects, and antibiofilm activity. Overall, the diagram highlights the multifactorial nature of AgNP action and the contribution of plant‐derived phytochemicals as reducing and stabilizing agents during nanoparticle formation. The frequency of reporting is provided to reflect how often each mechanism was described in the included studies and should not be interpreted as a formal measure of the quality or certainty of evidence.

Of the 63 included studies, 57 reported at least one proposed mechanism, whereas six did not provide any mechanistic explanation. The most commonly described effects were related to membrane disruption and increased permeability (39 reports) and ROS generation/oxidative stress (32 reports). These mechanisms were often accompanied by damage to proteins, lipids, or DNA. Direct DNA or protein injury, or inhibition of replication and/or translation, was explicitly reported in 17 studies, while protein damage or inhibition of replication‐related processes was described in 14 reports. In contrast, resistance‐ and transport‐related mechanisms, such as efflux pump inhibition, porin suppression, or interference with the respiratory chain, were rarely reported. Only isolated reports described lipid peroxidation, apoptosis, or biofilm‐specific mechanisms (Table 1B). The summary of these mechanisms is presented in Figure 2.

### 3.7 Toxicity

The evidence on toxicity was limited but informative: 30 of the 63 studies reported at least one toxicity endpoint, whereas 33 did not provide toxicity data. Among the 30 studies with available data, 16 assessed cytotoxicity against cancer or other sensitive cell lines, most commonly MCF‐7 (10 studies) and A549 (five studies), followed by AGS, HepG2, HCT116, COLO205, B16F10, and a single brine shrimp lethality assay. Reported effects ranged from strong cytotoxicity (IC50 values as low as 3.16 µg/mL and an LC50 of 14.12 µg/mL) to more moderate activity, with one study reporting a substantially higher IC50 of 42.19 mg/mL. In parallel, 14 studies reported low or no toxicity, or clear biocompatibility, in normal systems, including HTECs, human skin fibroblasts, L929, HFF, Vero, RBCs/PBMCs, rBMSCs, and hPDLCs. Reported outcomes included nontoxic profiles, low hemolysis, and no significant cytotoxicity (Table 1B).

### 3.8 Antibacterial Results

The antibacterial results are summarized in Table 2. Inhibition zone data were reported in 28 studies. The most common pattern observed was a larger inhibition zone for green‐synthesized AgNPs compared with the plant extract (11 records), followed by activity similar to that of antibiotics (nine records) and smaller inhibition zones than antibiotics (eight records).

**Table 2:** Antibacterial effects of green‐synthesized AgNPs on *Acinetobacter baumannii*.

Study ID	Comparator(s)	Inhibition zone of AgNPs	Comparator inhibition zone	IZ interpretation	MIC of AgNPs (µg/mL)	Comparator MIC (µg/mL)	MIC interpretation	MBC of AgNPs (µg/mL)	Comparator MBC (µg/mL)	MBC interpretation
S1	EX	*Thymus vulgaris*: 6 mm; *Mentha piperita*: 8 mm; *Zingiber officinale*: 6 mm	EX: 6 mm	*T. vulgaris*: similar to EX; *M. piperita*: larger than EX; *Z. officinale*: similar to EX	NA	NA	NA	NA	NA	NA
S2	EX, MEM, carbapenem, chemical AgNPs	19 mm	EX: 4 mm; MEM: 25 mm	Larger than EX; smaller than MEM	NA	NA	NA	NA	NA	NA
S3	EX, carbapenem, chemical AgNPs	25 mm at 200 µg/mL	EX: 14 mm; carbapenem: 27 mm; chemical AgNPs: 19 mm at 200 µg/mL	Larger than EX and chemical AgNPs; similar to carbapenem	2	EX: 125, Carbapenem: 1, Chemical AgNP: 31.25	Lower MIC values than EX and chemical AgNPs; higher MIC values than carbapenem	2	EX: 250; carbapenem: 1; chemical AgNPs: 31.25	Lower MBC values than EX and chemical AgNPs; higher MBC values than carbapenem
S4	NA	NA	NA	NA	78	NA	NA	312	NA	NA
S5	NA	NA	NA	NA	125	NA	NA	NA	NA	NA
S6	NA	11–14 mm	NA	NA	67.5	NA	NA	NA	NA	NA
S7	Aqueous EX, methanolic EX, AMP 10 µg/mL	23 mm	Aqueous EX: 10 mm; methanolic EX: 15 mm; AMP: 28 mm	Larger than EX; smaller than, but close to, AMP	NA	NA	NA	NA	NA	NA
S8	EX	25 mm	EX: 0 mm	Larger than EX	NA	NA	NA	NA	NA	NA
S9	COL 10 µg	12–17 mm	COL: 14 mm	Similar to COL	NA	NA	NA	NA	NA	NA
S10	CHL	NA	NA	NA	300	CHL: ~500	Lower MIC values than CHL	620	CHL: ~1000	Lower MBC values than CHL
S11	AgNO_3_	*Eragrostis tef*: 15–20 mm; *Vitellaria paradoxa*: 12–19 mm	AgNO_3_: 14–19 mm	Similar to AgNO_4_	NA	NA	NA	NA	NA	NA
S12	TIG, methanol only	21.04 mm	TIG: 20.63 mm; methanol only: 0 mm	Similar to, or slightly larger than, TIG	NA	NA	NA	NA	NA	NA
S13	NA	~11 mm (at 10 µL) to ~16 mm (at 50 µL)	NA	NA	NA	NA	NA	NA	NA	NA
S14	NA	31 mm at 100 µg/mL	NA	NA	NA	NA	NA	NA	NA	NA
S15	NA	NA	NA	NA	Standard strain: 1600; clinical strain: 3200	NA	NA	NA	NA	NA
S16	EX	11 mm at 1.5 mL; 5.5 mm at 3.5 mL; 7.4 mm at 5.5 mL	EX: 0 mm	Larger than EX	NA	NA	NA	NA	NA	NA
S17^a^	EX, COL, chemical AgNPs, untreated	NA	NA	NA	NA	NA	NA	NA	NA	NA
S18	AgNO_3_	~13 mm at 100 µg	AgNO_3_: ~15 mm	Larger than AgNO_3_	~50	NA	NA	~100	NA	NA
S19	VAN 30 µg, solvent	21.5 mm	VAN: 21.2 mm; solvent: 6 mm	Similar to VAN	NA	NA	NA	NA	NA	NA
S20	TET	15.33 mm	TET: 15.33 mm	Similar to TET	16	TET: 0.5	Higher MIC values than TET	16	TET: 2	Higher MBC values than TET
S21	NA	NA	NA	NA	25–200	NA	NA	50–200	NA	NA
S22	NA	NA	NA	NA	Clinical isolate: 0.32; standard strain: 0.64	NA	NA	Clinical isolate: 1.27; standard strain: 1.27	NA	NA
S23	AMP	NA	NA	NA	AgNP1: 125; AgNP2: 250; AgNP3: 500	AMP: 125	AgNP1 (small size, 1–10 nm) showed similar MIC values to AMP; the other formulations showed higher MIC values than AMP	NA	NA	NA
S24	EX, PEN 10 µg, STR 10 µg	17 mm	EX: 11 mm; PEN: 12 mm; STR: NA	Larger than EX and PEN	2.5	EX: 10; PEN: NA; STR: 4	Lower MIC values than EX and STR	NA	EX: 10; PEN: NA; STR: NA	Similar to EX
S25	GEN, water	*A. baumannii*: 16.8 mm; MRAB: 17.7 mm at 360 µg/mL	GEN: *A. baumannii* 16 mm; MRAB 6 mm; water: *A. baumannii* 6 mm; MRAB 6 mm	At 360 µg/mL: larger than GEN against MRAB; similar to GEN against *A. baumannii*	*A. baumannii*: 5.6, MRAB: 5.7	NA	NA	*A. baumannii*: 22.5; MRAB: 11.25	NA	NA
S26	AgNO_3_	NA	NA	NA	< 19.53 μM	AgNO_3_: 7.81	NA	< 19.53 μM	NA	NA
S27	NA	26 mm at 100 µg/mL	NA	NA	NA	NA	NA	NA	NA	NA
S28	EX	NA	NA	NA	Standard strain: 0.11; clinical isolate: 0.89	EX: > 4000	Lower MIC values than EX	Standard strain: 0.22; clinical isolate: 14	NA	NA
S29	AMP	NA	NA	NA	125	AMP: 125	Similar MIC values to AMP	NA	NA	NA
S30	EX, COL, water	14 mm	EX: 0 mm; COL: 15 mm; water: 0 mm	Larger than EX; similar to COL	32	EX: NA, COL: 2	Higher MIC values than COL	64	EX: NA; COL: 4	Higher MBC values than COL
S31	EX, CIP 5 µg/mL, VAN 30 µg/mL	16.2 mm at 2 mg/mL	EX: 15.2 mm; CIP: 21.2 mm; VAN: 21.5 mm	Similar to EX; smaller than CIP and VAN	1000	EX: 1500	Slightly lower MIC values than EX	250	EX: 250	Similar to EX
S32	NA	13 mm at 1000 µg/disk	NA	NA	NA	NA	NA	NA	NA	NA
S33	EX, CIP	NA	NA	NA	7.8	EX: > 4000, CIP: 0.25	Lower MIC values than EX; higher MIC values than CIP	62.5	NA	NA
S34	EX, CIP	NA	NA	NA	3.12	EX: > 4000, CIP: 0.26	Lower MIC values than EX; higher MIC values than CIP	0.39	NA	NA
S35	CIP	NA	NA	NA	*Mentha pulegium*: clinical MDR isolate: 0.4; reference strain: 0.8; *Crocus caspius*: clinical MDR isolate: 16; reference strain: 2	CIP: 0.25	Higher MIC values than CIP	*M. pulegium*: Clinical MDR isolate: 12.5, Reference strain: 6; *C. caspius*: Clinical MDR isolate: 36, Reference strain: 5	NA	NA
S36	EX, AgNO_3_	8.7 mm	EX: 5.1 mm; AgNO_3_: 8.7 mm	Larger than EX; similar to AgNO_3_	NA	NA	NA	NA	NA	NA
S37	NA	NA	NA	NA	62.5	NA	NA	250	NA	NA
S38	EX, TET	NA	NA	NA	62.5	EX: 250, TET: 125	Lower MIC values than EX; higher MIC values than TET	125	EX: > 500; TET: 250	Lower MBC values than EX and TET
S39	NA	NA	NA	NA	1.55	NA	NA	3.12	NA	NA
S40	GEN	*Salvadora persica* NP: no activity; *Caccinia macranthera* NP: *A. baumannii* (susceptible), 14 mm; *A. baumannii* (resistant), 11 mm	*A. baumannii* (susceptible): 17 mm; *A. baumannii* (resistant): 25 mm	*S. persica* NP: no activity; *C. macranthera* NP: smaller than GEN	*S. persica* NP: no activity; *C. macranthera* NP: *A. baumannii* (susceptible): 62.5; *A. baumannii* (resistant): 62.5	NA	NA	*S. persica* NP: No activity, *C. macranthera* NP: *A. baumannii* (susceptible): 31.25, *A. baumannii* (resistant): 126	NA	NA
S41	Water, AgNO_3_, methanol, chemical AgNPs, EX, blank media, AMP, VAN, GEN, TET	11.5–21 mm	Water, AgNO_3_, methanol, chemical AgNPs, EX, and blank media: 0 mm; VAN: 9 mm; GEN: 14 mm; TET: 22 mm; AMP: 0 mm	Larger than EX, chemical AgNPs, and AMP; similar to VAN, GEN, and TET	1.5	NA	NA	3.125	NA	NA
S42	EX	NA	NA	NA	2	EX: > 64	Lower MIC values than EX	NA	NA	NA
S43	EX, AgNO_3_, CIP	NA	NA	NA	*Eucomis autumnalis* NP: 60, *Sclerocarya birrea* NP: 131	*E. autumnalis* EX: > 8000; *S. birrea* EX: 4000; AgNO_3_: 40; CIP: 0.039	AgNPs from both plants showed lower MIC values than EX, similar MIC values to AgNO_3_, and higher MIC values than CIP	NA	NA	NA
S44	NA	NA	NA	NA	78–312	NA	NA	156–625	NA	NA
S45	NA	NA	NA	NA	64–128	NA	NA	64 to > 512	NA	NA
S46	MEM 10 µg	~12–19 mm	~19 mm	Similar to MEM	NA	NA	NA	NA	NA	NA
S47	AgNO_3_ (1 mM/mL), GEN 30 µg/mL	10–13 mm	AgNO_3_: 11 mm; GEN: 20 mm	Similar to AgNO_3_; smaller than GEN	NA	NA	NA	NA	NA	NA
S48	EX	NA	NA	NA	COL/IMP‐resistant *A. baumannii*: 4	COL/IMP‐resistant *A. baumannii*: > 64	Lower MIC values than EX	NA	NA	NA
S49	EX	NA	NA	NA	COL/IMP‐resistant *A. baumannii*: 2–8	COL/IMP‐resistant *A. baumannii*: > 64	NA	NA	NA	NA
S50	EX, RIF, GEN	12 mm	EX: NA; RIF: NA; GEN: 11 mm	Similar to GEN	1000	EX: > 16,000; RIF: 3.9; GEN: 7.8	Lower MIC values than EX; higher MIC values than RIF and GEN	1000	EX: > 16,000; RIF: 31.25; GEN: 15.63	Lower MBC values than EX; higher MBC values than RIF and GEN
S51	EX	1.2–2.1 mm	1 mm	Similar to EX	NA	NA	NA	NA	NA	NA
S52	AgNO_3_, antibiotic	7.85–13.35 mm	AgNO_3_: 23.6 mm; antibiotic: 16.94 mm	Smaller than AgNO_3_ and the antibiotic comparator	8	NA	NA	32	NA	NA
S53	NA	MTCC 1926: 16 mm; SN15: 11 mm	NA	NA	MTCC 1926: 6.25; SN15: 0.78125	NA	NA	MTCC 1926: 12.5; SN15: 1.5625	NA	NA
S54	EX, COL	21.62 mm	NA	NA	62.5	EX: > 500; COL: 1	Lower MIC values than EX; higher MIC values than COL	250	EX: > 500; COL: 2	Lower MBC values than EX; higher MBC values than COL
S55	CAZ	NA	NA	NA	MIC90: 4.6 (MIC50: NA)	CAZ: 1	Higher MIC values than CAZ	0.36	CAZ: 8	Lower MBC values than CAZ
S56	NA	1.6–1.8 mm	NA	NA	NA	NA	NA	NA	NA	NA
S57	EX, AgNO_3_	30 mm	EX: 17 mm; AgNO_3_: 7 mm	Larger than EX and AgNO_3_	10–21	NA	NA	NA	NA	NA
S58	Antibiotics	16–20 mm	0 mm	Larger than antibiotics	NA	NA	NA	NA	NA	NA
S59	AMP	3.35–11.45 mm	15 mm	Smaller than AMP	NA	NA	NA	NA	NA	NA
S60	NA	NA	NA	NA	50–400	NA	NA	NA	NA	NA
S61	EX, CIP	NA	NA	NA	Reference strain: 0.1; clinical isolates: 4	Reference strain: EX: > 4000; CIP: 0.25; Clinical strain: NA	Lower MIC values than EX and CIP	Reference strain: 0.2; clinical isolates: 0.1	NA	NA
S62	COL	11 mm at 3 mg/mL	NA	NA	0.09	COL: 2	Lower MIC values than COL	0.36–0.72	COL: 1–2	Lower MBC values than COL
S63	NA	11 mm at 3 mg/mL	NA	NA	250	NA	NA	NA	NA	NA

*Note:* The study IDs used in this table correspond to those presented in Table 1A. For clarity and simplicity, standard deviation values were omitted, and only the primary data are presented.

Abbreviations: AMP, ampicillin; CAZ, ceftazidime; CHL, chloramphenicol; CIP, ciprofloxacin; COL, colistin; EX, plant extract; GEN, gentamicin; IMP, imipenem; IZ, inhibition zone; MBC, minimum bactericidal concentration; MDR, multidrug resistant; MEM, meropenem; MIC, minimum inhibitory concentration; MRAB, multidrug‐resistant *Acinetobacter baumannii*; MTCC, microbial type culture collection; NA, not available; NP, nanoparticle; PEN, penicillin; RIF, rifampin; STR, streptomycin; TET, tetracycline; TIG, tigecycline; VAN, vancomycin.

^a^ The antibacterial effect in this study was evaluated only by CFU counting, which showed complete bactericidal activity (no viable cells) after 3 h of incubation. The activity was comparable to that of chemical AgNPs and colistin, and greater than that of the EX.

Minimum inhibitory concentration (MIC) values were reported in 18 studies. The most frequent finding was lower MIC values for green‐synthesized AgNPs than for the extract (13 records). Comparisons with antibiotics were more variable, with five records showing lower MIC values than antibiotics and 12 records showing higher MIC values than antibiotics.

Minimum bactericidal concentration (MBC) was reported in 11 studies, with four records showing lower MBC values than antibiotics, four showing lower values than the extract, and five showing higher values than antibiotics (Table 2). Note that some studies included more than one comparator, which is why the number of records and studies differ.

Biofilm inhibition was reported in only three studies. One study reported an approximately twofold reduction in biofilm formation (S5), another showed a 2.66‐fold reduction (S6), and the third reported 36.33%–80.07% biofilm inhibition and 28.04%–71.95% biofilm eradication at sub‐MIC concentrations (S45).

In one study (S17), the antibacterial effect was evaluated solely by CFU counting, which demonstrated complete bactericidal activity (no viable cells) after 3 h of incubation. The activity was comparable to that of chemically synthesized AgNPs and colistin, and greater than that of the extract.

## 4 Discussion

*A. baumannii* is a major cause of MDR hospital infections, and its ability to persist, resist antibiotics, and form biofilms makes treatment difficult (Sabzi et al. 2024). In this context, the present systematic review examines whether plant‐mediated AgNPs can serve as a useful alternative or adjunct against MDR *A. baumannii*. Overall, the findings show that green‐synthesized AgNPs are generally antibacterial, often more effective than plant extracts and sometimes comparable to antibiotics, although their activity varies substantially depending on synthesis conditions and nanoparticle properties.

The broad eligibility criteria adopted in this review were intentional, as the primary aim was to provide a comprehensive overview of the current evidence on plant‐mediated AgNPs against *A. baumannii* rather than to estimate a single pooled effect size. Considerable heterogeneity was expected because green synthesis is inherently influenced by plant species, extraction procedures, nanoparticle physicochemical characteristics, bacterial strains, and experimental protocols. Instead of limiting the review to narrowly defined conditions, the included studies were systematically grouped and narratively synthesized according to common themes, including nanoparticle characteristics, antibacterial activity, mechanisms of action, toxicity, and antibiofilm effects. This approach allowed the identification of recurring trends, factors associated with antibacterial performance, and important knowledge gaps, while also highlighting the need for greater methodological standardization in future studies. Therefore, although direct quantitative comparisons between studies should be interpreted with caution, the overall evidence provides a valuable and comprehensive understanding of the current state of the field.

Most of the included studies were conducted in Asia, especially in regions where traditional medicine and plant‐based knowledge are well established. One possible reason is that these research settings naturally support green‐synthesis studies. Another is that testing the antibacterial effect of green‐synthesized nanoparticles is relatively simple compared with many other complex experimental approaches, so researchers may be more likely to adopt this approach.

The predominance of Lamiaceae, Asteraceae, and Fabaceae may be related to the wide availability and rich phytochemical composition of these plant families, which are known to contain high levels of phenolics, flavonoids, terpenoids, and other bioactive metabolites involved in the reduction and stabilization of AgNPs (Mustapha and Misni 2022). This pattern is consistent with the broader green‐synthesis literature, which emphasizes that plant biomolecules function simultaneously as reducing and stabilizing agents, allowing low‐cost, low‐solvent, and relatively scalable fabrication under mild conditions (F. Arshad et al. 2024; Huq et al. 2022; Thomas et al. 2024). In practical terms, this matters because many developing or resource‐conscious settings need antimicrobial technologies that are less energy intensive and less dependent on hazardous chemical reducers than conventional nanoparticle synthesis routes. The fact that most studies included in this review used dried plant material, with maceration and decoction as the dominant extraction methods and water as the most common solvent, also reinforces the real‐world appeal of the field, even though it simultaneously introduces variability in phytochemical yield and extract composition.

From a nanomaterials perspective, the physicochemical profiles of the nanoparticles in this review are encouraging yet heterogeneous. Most AgNPs were smaller than 100 nm, and the majority were below 50 nm, which is relevant because smaller particles generally provide a larger surface area‐to‐volume ratio, more frequent bacterial contact, and a greater likelihood of silver‐ion release and membrane interaction (Silva‐Holguín et al. 2024). Spherical morphology dominated, while zeta potential was reported in only a minority of studies, most often in the moderately to strongly negative range, and many nanoparticles were described as face‐centered cubic crystalline structures. These characteristics matter because the synthesis method, particle size, morphology, surface charge, and capping layer can strongly shape antibacterial potency, colloidal stability, and biological interactions (Sati et al. 2025; Thomas et al. 2024). The studies suggest that the field is now moving beyond basic proof of concept toward better optimization, but characterization is still not fully consistent or standardized. In some reports, incomplete zeta potential data, signs of agglomeration, and different size measurements across methods suggest that some nanoparticles were probably less stable than their antibacterial results alone imply. This may also explain why similar plant sources sometimes showed different levels of antibacterial activity across studies.

The results show that green‐synthesized AgNPs possess genuine antibacterial activity, although the strength of this effect varies across studies. In this review, green AgNPs more often produced larger inhibition zones than the corresponding plant extracts, and MIC values were usually lower than those of the extracts, which supports the interpretation that nanoscale silver provides antibacterial potency beyond phytochemical exposure alone. At the same time, comparison with antibiotics was mixed: in some studies AgNPs were comparable to or better than conventional drugs, whereas in more studies they were less active. This is not a failure of the platform; rather, it underscores that AgNPs should not be treated as a uniform antibiotic substitute. Their performance depends on particle size, concentration, surface chemistry, and the bacterial model used, as well as the comparator antibiotic and the assay format. The literature also indicates that AgNPs can enhance or restore antibiotic activity when used as adjuvants, which represents a particularly important translational strategy for treating MDR Gram‐negative infections (Rodrigues et al. 2024). Recent work has shown that nanoparticle‐antibiotic interactions can be synergistic, additive, or antagonistic, and that the desired outcome is synergy, especially where outer‐membrane permeability and efflux contribute to resistance (Abdullah et al. 2024; Crisan et al. 2024). The present review aligns with that framework: green AgNPs appear more promising as antibiotic‐sparing enhancers or resistance modifiers than as stand‐alone replacements for optimized antimicrobials.

The mechanisms reported in these studies are biologically plausible and are consistent with the current understanding of how AgNPs act on bacteria. Membrane and cell–wall disruption (39 reports) and ROS generation/oxidative stress (32 reports) were the most frequently reported mechanisms, followed by damage to proteins, lipids, and nucleic acids, as well as interference with respiratory activity. These mechanisms are consistent with the current understanding of AgNP action, in which direct membrane contact, Ag^+^ release, intracellular redox imbalance, and interference with essential macromolecules collectively produce rapid bactericidal effects (Rodrigues et al. 2024; Sati et al. 2025). The limited reporting of efflux inhibition, porin downregulation, and respiratory‐chain interference suggests that these mechanisms likely occur but are experimentally harder to confirm and are therefore often inferred from indirect rather than direct evidence. One novel implication of the present review is that the phytochemical corona created during green synthesis may contribute to the multifactorial activity by improving dispersion, mediating adhesion to bacterial surfaces, and possibly modulating gene expression or membrane transport. This may help explain why green AgNPs sometimes perform better than chemically synthesized particles in otherwise similar antibacterial contexts, even though the underlying chemistry is not yet fully resolved.

Biofilm inhibition remains the most important unresolved therapeutic challenge in the literature. Only four studies assessed biofilms, and only three reported explicit inhibition or eradication outcomes, despite the central role of biofilms in *A. baumannii* persistence and device‐associated infections. This scarcity is disappointing but unsurprising, because biofilms are structurally complex, phenotypically heterogeneous, and intrinsically tolerant to antimicrobials due to matrix shielding, reduced metabolic activity, quorum‐sensing coordination, and heterogeneous diffusion barriers (Rajangam and Narasimhan 2024). The few studies available are nonetheless encouraging, showing measurable reductions in biofilm formation and even sub‐MIC eradication in one study. These findings suggest that green AgNPs may be especially useful when the clinical problem is not simply planktonic growth inhibition but persistent colonization of catheters, tubing, or wound beds. That said, biofilm data must be interpreted cautiously because assays differ widely in inoculum density, maturation time, surface type, readout method, and whether they assess biofilm inhibition or eradication. In future work, standardized mature‐biofilm models, microscopy‐based confirmation, and viability assays should be combined to determine whether the apparent antibiofilm activity is sufficiently robust for translational development.

When the findings are compared with conventional antibiotics, the main point is that these nanoparticles can be effective, but not always to the same extent as standard drugs. Several included studies showed that green AgNPs were comparable to clinically used antibiotics, including colistin. This is important because colistin remains one of the last‐line agents for CRAB, yet its use is constrained by nephrotoxicity, incomplete efficacy, and the emergence of resistance (Kubin et al. 2025). Nonetheless, the present review also shows that many AgNP formulations still underperform compared with antibiotics in terms of MIC or MBC. That inconsistency probably reflects not only bacterial phenotype and comparator potency, but also assay limitations. DD and agar well diffusion can underestimate nanoparticle activity because diffusion through agar depends on particle size, surface coating, aggregation state, and ionic release kinetics, whereas broth microdilution may better capture contact‐dependent killing (Chung et al. 2023). The methodological diversity in the literature, therefore, makes direct comparisons between studies difficult. Even so, the overall pattern supports the idea that green AgNPs may reduce antibiotic burden by potentiating existing drugs, lowering effective doses, and potentially disrupting resistance mechanisms rather than replacing antibiotics outright.

Safety remains an important issue because the toxicity data are limited and insufficient to fully assess the clinical suitability of these nanoparticles. About half of the included studies provided no toxicity data, and among those that did, the findings ranged from clear biocompatibility to substantial cytotoxicity in cancer or sensitive cell models. This duality is not surprising because the biological effects of AgNPs are dose‐dependent, strongly influenced by surface chemistry, and often shared between bacterial and mammalian membranes (Liao et al. 2019). The broader AgNP literature emphasizes that the synthesis method strongly influences purity, stability, and toxicity, and that plant‐derived capping layers can sometimes improve biocompatibility while retaining antimicrobial activity (Sati et al. 2025; Thomas et al. 2024). In the present review, some studies reported nontoxic profiles in normal human cells, red blood cells, or fibroblasts, whereas others showed marked cytotoxicity at relatively low concentrations. The practical implication is that antimicrobial potency alone is insufficient for clinical translation. Future development will require systematic toxicity profiling in relevant normal cell types, hemocompatibility studies, inflammatory profiling, and in vivo pharmacokinetic, biodistribution, and safety studies. Without this, even highly active formulations will remain difficult to translate beyond the bench.

There are some important limitations of the current study. First, although the review is comprehensive within its eligibility criteria, the included literature was highly heterogeneous, and the absence of a meta‐analysis means the findings are necessarily descriptive rather than quantitative. Second, the overall risk of bias was moderate across all studies, and many reports lacked detailed information on randomization, replication, concentration selection, or outcome measurement. Third, toxicity data were absent in a large proportion of studies, and almost none of the included experiments addressed pharmacokinetics, in vivo efficacy, or immunological safety. Fourth, biofilm experiments were scarce, and susceptibility testing often relied on diffusion‐based methods that are suboptimal for nanoparticle evaluation. Finally, the exclusive focus on pure plant‐mediated AgNPs, while scientifically justified to reduce confounding, also excluded potentially informative hybrid systems that might have stronger translational value.

Overall, this systematic review shows that plant‐mediated AgNPs have credible and reproducible antibacterial activity against *A. baumannii*, with the strongest evidence pointing to membrane disruption and oxidative stress as the dominant mechanisms of action, while evidence for biofilm inhibition remains limited. The novelty of the present work lies in its focus on pure green‐synthesized AgNPs specifically against *A. baumannii*, its integration of physicochemical and biological outcomes, and its critical comparison of antibacterial potency, toxicity, and methodological quality across 63 studies. The findings support continued development of green AgNPs as antibiofilm and antibiotic‐adjuvant platforms, particularly in settings where multidrug resistance is common and access to expensive novel agents is limited. Future work should prioritize standardized synthesis protocols, rigorous particle characterization, mature‐biofilm assays, clinically relevant MDR/XDR isolates, direct comparisons with last‐line antibiotics, and in vivo safety studies. If these steps are taken, green AgNPs could evolve from a promising laboratory strategy into a practical nanobiotechnological tool for combating difficult *A. baumannii* infections.

## Author Contributions

**Shahla Shahbazi:** conceptualization, investigation, writing – original draft, validation, writing – review and editing. **Kimia Rafieibuzhani:** investigation, methodology, data curation, writing – review and editing, writing – original draft. **Keyghobad Ghadiri:** writing – original draft, writing – review and editing, conceptualization. **Yadollah Bahrami:** writing – original draft, writing – review and editing, software, formal analysis. **Roya Chegene Lorestani:** writing – original draft, writing – review and editing, investigation, methodology, validation, data curation. **Maryam Meskini:** writing – original draft, writing – review and editing, validation, investigation. **Samira Sabzi:** writing – original draft, writing – review and editing, investigation. **Mosayeb Rostamian:** conceptualization, writing – original draft, writing – review and editing, validation, investigation, project administration, formal analysis, software, supervision.

## Ethics Statement

The authors have nothing to report.

## Consent

The authors have nothing to report.

## Conflicts of Interest

None declared.

## Use of AI Tools

ChatGPT (OpenAI, GPT‐5.4) was used exclusively to improve the clarity, fluency, and readability of the manuscript. Adobe Illustrator 2020 and ChatGPT were also used to assist with figure preparation and visualization. No AI tools were used for data collection, data analysis, interpretation of results, or generation of scientific conclusions. The study design, results, and interpretations are entirely the authors' own work.

## Supporting information


Supporting File 1

Supporting File 2

## Data Availability

All data related to this study are presented in the text, tables, and figures of the article.

## References

[mbo370378-bib-0001] Abdellatif, A. A. H. , S. S. Alhathloul , A. Aljohani , et al. 2022. “Green Synthesis of Silver Nanoparticles Incorporated Aromatherapies Utilized for Their Antioxidant and Antimicrobial Activities Against Some Clinical Bacterial Isolates.” Bioinorganic Chemistry and Applications 2022: 2432758. 35449714 10.1155/2022/2432758 PMC9017581

[mbo370378-bib-0002] Abdullah , T. Jamil , M. Atif , et al. 2024. “Recent Advances in the Development of Metal/Metal Oxide Nanoparticle and Antibiotic Conjugates (MNP‐Antibiotics) to Address Antibiotic Resistance: Review and Perspective.” International Journal of Molecular Sciences 25: 8915. 39201601 10.3390/ijms25168915 PMC11354832

[mbo370378-bib-0003] Abdussalam‐Mohammed, W. , K. Edbey , H. E. Farhat , P. Shah , S. S. Shamsi , and A. Bhattarai . 2025. “Facile Green Synthesis of Novel AgNPs Using *Hyoscyamus* Leaf Extract as Capping Agent: Characterization and Their Potential Antibacterial Activities.” Inorganic Chemistry Communications 173: 113893.

[mbo370378-bib-0004] Abootalebi, S. N. , S. M. Mousavi , S. A. Hashemi , E. Shorafa , N. Omidifar , and A. Gholami . 2021. “Antibacterial Effects of Green‐Synthesized Silver Nanoparticles Using *Ferula asafoetida* Against *Acinetobacter baumannii* Isolated From the Hospital Environment and Assessment of Their Cytotoxicity on the Human Cell Lines.” Journal of Nanomaterials 2021: 6676555.

[mbo370378-bib-0005] Ajlouni, A.‐W. , E. H. Hamdan , R. A. E. Alshalawi , et al. 2022. “Green Synthesis of Silver Nanoparticles Using Aerial Part Extract of the *Anthemis pseudocotula* Boiss. Plant and Their Biological Activity.” Molecules 28: 246. 36615440 10.3390/molecules28010246 PMC9822267

[mbo370378-bib-0006] Akar, Z. , S. Akay , N. Ejder , and A. Özad Düzgün . 2024. “Determination of the Cytotoxicity and Antibiofilm Potential Effect of *Equisetum arvense* Silver Nanoparticles.” Applied Biochemistry and Biotechnology 196: 909–922. 37273097 10.1007/s12010-023-04587-7

[mbo370378-bib-0007] Akay, S. , G. Yüksel , and A. Özad Düzgün . 2024. “Investigation of Antibiofilm and Antibacterial Properties of Green Synthesized Silver Nanoparticles From Aqueous Extract of *Rumex* sp.” Applied Biochemistry and Biotechnology 196: 1089–1103. 37329410 10.1007/s12010-023-04592-w

[mbo370378-bib-0008] Akther, T. , S. Priya , S. K. Sah , K. M. Shahanbaj , and S. Hemalatha . 2019. “Ta‐AgNps Are Potential Antimicrobial Resistance Breakers.” Journal of Nanostructures 9: 376–383.

[mbo370378-bib-0009] Alhamdi, H. W. , F. A. Mokhtar , F. L. Ridouane , et al. 2024. “Computational Metal–Flavonoids Complexes Presentation of Greenly Synthesized Silver Nanoparticles Combined Flavonoids From *Lens culinaris* L. as Anticancer Agents Using BcL‐2 and IspC Proteins.” Artificial Cells, Nanomedicine, and Biotechnology 52: 529–550. 39462870 10.1080/21691401.2024.2420414

[mbo370378-bib-0010] Alharbi, F. A. , and A. A. Alarfaj . 2020. “Green Synthesis of Silver Nanoparticles From *Neurada procumbens* and Its Antibacterial Activity Against Multi‐Drug Resistant Microbial Pathogens.” Journal of King Saud University‐Science 32: 1346–1352.

[mbo370378-bib-0011] Alharbi, N. S. , J. M. Khaled , K. Alanazi , S. Kadaikunnan , and A. S. Alobaidi . 2023. “Biosynthesis of Silver Nanoparticles (Ag‐Nps) Using *Senna alexandrina* Grown in Saudi Arabia and Their Bioactivity Against Multidrug‐Resistant Pathogens and Cancer Cells.” Saudi Pharmaceutical Journal 31: 911–920. 37234348 10.1016/j.jsps.2023.04.015 PMC10205756

[mbo370378-bib-0012] Almudhafar, S. M. A. , and M. A. Al‐Hamdani . 2022. “Antibacterial and Anticancer Effects of Silver Nanoparticles Synthesised Using *Eragrostis tef* and *Vitellaria paradoxa* Seeds Extract.” Basrah Journal of Agricultural Sciences 35: 132–159.

[mbo370378-bib-0013] Al‐Otibi, F. O. , M. T. Yassin , A. A. Al‐Askar , and K. Maniah . 2023. “Green Biofabrication of Silver Nanoparticles of Potential Synergistic Activity With Antibacterial and Antifungal Agents Against Some Nosocomial Pathogens.” Microorganisms 11: 945. 37110368 10.3390/microorganisms11040945 PMC10144991

[mbo370378-bib-0014] AlSalhi, M. S. , S. Devanesan , A. A. Alfuraydi , et al. 2016. “Green Synthesis of Silver Nanoparticles Using *Pimpinella anisum* Seeds: Antimicrobial Activity and Cytotoxicity on Human Neonatal Skin Stromal Cells and Colon Cancer Cells.” International Journal of Nanomedicine 11: 4439–4449. 27660438 10.2147/IJN.S113193 PMC5019319

[mbo370378-bib-0015] AlSalhi, M. S. , K. Elangovan , A. J. A. Ranjitsingh , P. Murali , and S. Devanesan . 2019. “Synthesis of Silver Nanoparticles Using Plant Derived 4‐*N*‐Methyl Benzoic Acid and Evaluation of Antimicrobial, Antioxidant and Antitumor Activity.” Saudi Journal of Biological Sciences 26: 970–978. 31303827 10.1016/j.sjbs.2019.04.001 PMC6600725

[mbo370378-bib-0016] Alsharari, S. S. , F. Alown , F. Ameen , and N. Majrashi . 2024. “Biogenic Silver Nanoparticles Synthesized Using Rose Petals: Potential to Inhibit Multidrug‐Resistant Bacterial Pathogens in Disabled Patients.” South African Journal of Botany 174: 405–409.

[mbo370378-bib-0017] AL‐Zubaidi, H. Y. , and A. F. Kamil . 2025. “Study of Optimum Conditions and Characterization for Green Synthesis of Silver Nanoparticles Using *Zamioculcas zamiifolia* Leaves Extract and Using Its Against Bacterial Pathogens.” Journal of Nanostructures 15: 1656–1666.

[mbo370378-bib-0018] Aman, S. , N. Kaur , D. Mittal , et al. 2023. “Novel Biocompatible Green Silver Nanoparticles Efficiently Eliminates Multidrug Resistant Nosocomial Pathogens and Mycobacterium Species.” Indian Journal of Microbiology 63: 73–83. 37188239 10.1007/s12088-023-01061-0 PMC10172440

[mbo370378-bib-0019] Arshad, F. , G. A. Naikoo , I. U. Hassan , et al. 2024. “Bioinspired and Green Synthesis of Silver Nanoparticles for Medical Applications: A Green Perspective.” Applied Biochemistry and Biotechnology 196: 3636–3669. 37668757 10.1007/s12010-023-04719-z PMC11166857

[mbo370378-bib-0020] Arshad, H. , M. Saleem , U. Pasha , and S. Sadaf . 2022. “Synthesis of *Aloe vera*‐Conjugated Silver Nanoparticles for Use Against Multidrug‐Resistant Microorganisms.” Electronic Journal of Biotechnology 55: 55–64.

[mbo370378-bib-0021] Baghayeri, M. , B. Mahdavi , Z. Hosseinpor‐Mohsen Abadi , and S. Farhadi . 2018. “Retracted: Green Synthesis of Silver Nanoparticles Using Water Extract of *Salvia leriifolia*: Antibacterial Studies and Applications as Catalysts in the Electrochemical Detection of Nitrite.” Applied Organometallic Chemistry 32: e4057.

[mbo370378-bib-0022] Bahrami, Y. , A. Darvishi , K. Ghadiri , et al. 2025. “Antibiotic‐Peptide Conjugates as Alternatives to Conventional Antibiotics for Treating Infections Caused by Hospital‐Associated ESKAPE Pathogens: A Systematic Review.” Microbial Pathogenesis 207: 107927. 40714270 10.1016/j.micpath.2025.107927

[mbo370378-bib-0023] Barabadi, H. , F. Mojab , S. Amidi , et al. 2024. “Phytofabrication, Characterization and Investigation of Biological Properties of *Punica granatum* Flower‐Derived Silver Nanoparticles.” Inorganic Chemistry Communications 170: 113515.

[mbo370378-bib-0024] Behdad, R. , M. Pargol , A. Mirzaie , S. Z. Karizi , H. Noorbazargan , and I. Akbarzadeh . 2020. “Efflux Pump Inhibitory Activity of Biologically Synthesized Silver Nanoparticles Against Multidrug‐Resistant *Acinetobacter baumannii* Clinical Isolates.” Journal of Basic Microbiology 60: 494–507. 32301139 10.1002/jobm.201900712

[mbo370378-bib-0025] Buapoon, S. , D. Khwannimit , P. Wintachai , and P. Rattanakit . 2021. “Naked‐Eye Copper (II) Sensing and Antibacterial Performance of Silver Nanoparticles Synthesized Using Butterfly Pea Aqueous Extract.” Nanotechnology for Environmental Engineering 6: 38.

[mbo370378-bib-0026] Ceylan, Ö. , and N. Hacıoğlu Doğru . 2025. “Biological Activities of Silver Nanoparticles Synthesized Using *Olea europaea* L. Leaves.” International Journal of Secondary Metabolite 12: 289–296.

[mbo370378-bib-0027] Charlz Nithin, J. , S. Ranjani , and S. Hemalatha . 2022. “ *Mimusops elengi* Flower‐Mediated Green Silver Nanoparticles Control *Staphylococcus aureus* and *Acinetobacter baumannii* .” Applied Biochemistry and Biotechnology 194: 3066–3081. 35347673 10.1007/s12010-022-03882-z

[mbo370378-bib-0028] Chegene Lorestani, R. , A. Shojaeian , and M. Rostamian . 2023. “Phenotypic, Genotypic, and Metabolic Resistance Mechanisms of ESKAPE Bacteria to Chemical Disinfectants: A Systematic Review and Meta‐Analysis.” Expert Review of Anti‐Infective Therapy 21: 1097–1123. 37674347 10.1080/14787210.2023.2256975

[mbo370378-bib-0029] Choi, J. , H. Jung , Y. Baek , et al. 2021. “Antibacterial Activity of Green‐Synthesized Silver Nanoparticles Using *Areca catechu* Extract Against Antibiotic‐Resistant Bacteria.” Nanomaterials 11: 205. 33466916 10.3390/nano11010205 PMC7830304

[mbo370378-bib-0030] Chung, E. , G. Ren , I. Johnston , et al. 2023. “Applied Methods to Assess the Antimicrobial Activity of Metallic‐Based Nanoparticles.” Bioengineering (Basel, Switzerland) 10: 1259. 38002383 10.3390/bioengineering10111259 PMC10669044

[mbo370378-bib-0031] Crisan, M. C. , S. L. Pandrea , L. Matros , T. Mocan , and L. Mocan . 2024. “In Vitro Antimicrobial Activity of Silver Nanoparticles Against Selected Gram‐Negative and Gram‐Positive Pathogens.” Medicine and Pharmacy Reports 97: 280–297. 39234464 10.15386/mpr-2750 PMC11370865

[mbo370378-bib-0032] Deonas, A. N. , L. M. S. Souza , G. J. S. Andrade , et al. 2024. “Green Synthesis of Silver Nanoparticle From *Anadenanthera colubrina* Extract and Its Antimicrobial Action Against ESKAPEE Group Bacteria.” Antibiotics (USSR) 13: 777. 10.3390/antibiotics13080777 PMC11352003 39200077

[mbo370378-bib-0033] Devanesan, S. , M. Jayamala , M. S. AlSalhi , S. Umamaheshwari , and A. J. A. Ranjitsingh . 2021. “Antimicrobial and Anticancer Properties of *Carica papaya* Leaves Derived Di‐Methyl Flubendazole Mediated Silver Nanoparticles.” Journal of infection and Public Health 14: 577–587. 33848887 10.1016/j.jiph.2021.02.004

[mbo370378-bib-0034] Doğan Çalhan, S. , and M. Gündoğan . 2020. “Biosynthesis of Silver Nanoparticles Using *Onosma sericeum* Willd. and Evaluation of Their Catalytic Properties and Antibacterial and Cytotoxic Activity.” Turkish Journal of Chemistry 44: 1587–1600. 33488255 10.3906/kim-2007-1 PMC7765767

[mbo370378-bib-0035] Dubey, R. K. , S. Shukla , and Z. Hussain . 2023. “Green Synthesis of Silver Nanoparticles; A Sustainable Approach With Diverse Applications.” Chinese Journal of Applied Physiology 39: e20230007. 38763765 10.62958/j.cjap.2023.007

[mbo370378-bib-0036] Ebrahimzadeh, M. A. , A. Naghizadeh , O. Amiri , M. Shirzadi‐Ahodashti , and S. Mortazavi‐Derazkola . 2020. “Green and Facile Synthesis of Ag Nanoparticles Using *Crataegus pentagyna* Fruit Extract (CP‐AgNPs) for Organic Pollution Dyes Degradation and Antibacterial Application.” Bioorganic Chemistry 94: 103425. 31740048 10.1016/j.bioorg.2019.103425

[mbo370378-bib-0037] El Megdar, S. , L. Fayzi , R. Elkheloui , et al. 2024. “Biological Synthesis of Silver Nanoparticles From *Lavandula mairei* Humbert: Antibacterial and Antioxidant Activities.” Current Microbiology 81: 151. 38647541 10.1007/s00284-024-03670-4

[mbo370378-bib-0038] El Megdar, S. , A. Laktib , R. Elkheloui , et al. 2025. “Optimizing *Lavandula mairei*‐Mediated Silver Nanoparticle Synthesis for Effective Inhibition and Eradication of Multidrug‐Resistant Bacterial Biofilms.” Colloids and Surfaces, A: Physicochemical and Engineering Aspects 714: 136544.

[mbo370378-bib-0039] Eroğlu, P. , and F. Valiyeva . 2025. “Investigation of Biological Activities of Silver Nanoparticles Synthesis From the Root Extract of Endemic *Onosma mutabilis* (*O. mutabilis*) Plant.” Spectroscopy Letters 58: 515–529.

[mbo370378-bib-0040] Gautam, D. , K. G. Dolma , B. Khandelwal , et al. 2023. “Green Synthesis of Silver Nanoparticles Using *Ocimum sanctum* Linn. and Its Antibacterial Activity Against Multidrug Resistant *Acinetobacter baumannii* .” PeerJ 11: e15590. 37529215 10.7717/peerj.15590 PMC10389072

[mbo370378-bib-0041] Geyesa, J. M. , T. B. Esho , B. A. Legesse , and A. S. Wotango . 2024. “Antibacterial and Antioxidant Potential Analysis of *Verbascum sinaiticum* Leaf Extract and Its Synthesized Silver Nanoparticles.” Heliyon 10: e24215. 38268826 10.1016/j.heliyon.2024.e24215 PMC10803913

[mbo370378-bib-0042] Ghosh, S. , S. Patil , M. Ahire , et al. 2012. “Synthesis of Silver Nanoparticles Using *Dioscorea bulbifera* Tuber Extract and Evaluation of Its Synergistic Potential in Combination With Antimicrobial Agents.” International Journal of Nanomedicine 7: 483–496. 22334779 10.2147/IJN.S24793 PMC3273981

[mbo370378-bib-0043] Hashemi, Z. , Z. M. Mizwari , S. R. Alizadeh , et al. 2023. “Anticancer and Antibacterial Activity Against Clinical Pathogenic Multi‐Drug Resistant Bacteria Using Biosynthesized Silver Nanoparticles With *Mentha pulegium* and *Crocus caspius* Extracts.” Inorganic Chemistry Communications 154: 110982.

[mbo370378-bib-0044] Hashemi, Z. , M. Mohammadyan , S. Naderi , et al. 2021. “Green Synthesis of Silver Nanoparticles Using *Ferula persica* Extract (Fp‐NPs): Characterization, Antibacterial, Antileishmanial, and In Vitro Anticancer Activities.” Materials Today Communications 27: 102264.

[mbo370378-bib-0045] Hashemi, Z. , M. Shirzadi‐Ahodashti , S. Mortazavi‐Derazkola , and M. A. Ebrahimzadeh . 2022. “Sustainable Biosynthesis of Metallic Silver Nanoparticles Using Barberry Phenolic Extract: Optimization and Evaluation of Photocatalytic, In Vitro Cytotoxicity, and Antibacterial Activities Against Multidrug‐Resistant Bacteria.” Inorganic Chemistry Communications 139: 109320.

[mbo370378-bib-0093] Heidarinia, H. , E. Tajbakhsh , Y. Bahrami , and M. Rostamian . 2025. “Design a Multi‐Epitope Vaccine Candidate Against *Acinetobacter baumannii* Using Advanced Computational Methods.” AMB Express 15: 103. 10.1186/s13568-025-01913-6. 40650846 PMC12255626

[mbo370378-bib-0046] Hou, T. , Y. Guo , W. Han , et al. 2023. “Exploring the Biomedical Applications of Biosynthesized Silver Nanoparticles Using *Perilla frutescens* Flavonoid Extract: Antibacterial, Antioxidant, and Cell Toxicity Properties Against Colon Cancer Cells.” Molecules 28: 6431. 37687260 10.3390/molecules28176431 PMC10490294

[mbo370378-bib-0047] Hudaya, I. R. , G. I. Mukti , A. Kasasiah , et al. 2024. “Eco‐Friendly Green Synthesis of Silver Nanoparticles Using *Toona sureni* (Blume) Merr. Leaf Extract and Evaluation of Its Antibacterial Activity Against Selected Clinical Isolates.” Chemistry Africa 7: 1311–1321.

[mbo370378-bib-0048] Hudaya, I. R. , V. C. Situmorang , F. A. Hardianto , et al. 2024. “Biofabrication of Silver Nanoparticles Using *Artocarpus heterophyllus* Leaves Extract: Characterization and Evaluation of Its Antibacterial, Antibiofilm, and Antioxidant Activities.” Journal of Crystal Growth 644: 127827.

[mbo370378-bib-0049] Huq, M. A. , M. Ashrafudoulla , M. M. Rahman , S. R. Balusamy , and S. Akter . 2022. “Green Synthesis and Potential Antibacterial Applications of Bioactive Silver Nanoparticles: A Review.” Polymers 14: 742. 35215655 10.3390/polym14040742 PMC8879957

[mbo370378-bib-0050] Ibrahim, S. , N. Al‐Saryi , I. M. S. Al‐Kadmy , and S. N. Aziz . 2021. “Multidrug‐Resistant *Acinetobacter baumannii* as an Emerging Concern in Hospitals.” Molecular Biology Reports 48: 6987–6998. 34460060 10.1007/s11033-021-06690-6 PMC8403534

[mbo370378-bib-0051] Iovleva, A. , V. G. Fowler, Jr. , and Y. Doi . 2025. “Treatment Approaches for Carbapenem‐Resistant *Acinetobacter baumannii* Infections.” Drugs 85: 21–40. 39607595 10.1007/s40265-024-02104-6 PMC11950131

[mbo370378-bib-0052] Jiang, X. , S. Khan , A. Dykes , E. Stulz , and X. Zhang . 2025. “Biogenic Synthesis of Silver Nanoparticles and Their Diverse Biomedical Applications.” Molecules (Basel, Switzerland) 30: 3104. 40807279 10.3390/molecules30153104 PMC12348928

[mbo370378-bib-0053] Khatami, M. , N. Zafarnia , M. Heydarpoor Bami , I. Sharifi , and H. Singh . 2018. “Antifungal and Antibacterial Activity of Densely Dispersed Silver Nanospheres With Homogeneity Size Which Synthesized Using Chicory: An In Vitro Study.” Journal de Mycologie Médicale 28: 637–644. 30100172 10.1016/j.mycmed.2018.07.007

[mbo370378-bib-0054] Khojasteh‐Taheri, R. , A. Ghasemi , Z. Meshkat , Z. Sabouri , M. Mohtashami , and M. Darroudi . 2023. “Green Synthesis of Silver Nanoparticles Using *Salvadora persica* and *Caccinia macranthera* Extracts: Cytotoxicity Analysis and Antimicrobial Activity Against Antibiotic‐Resistant Bacteria.” Applied Biochemistry and Biotechnology 195: 5120–5135. 36847984 10.1007/s12010-023-04407-y

[mbo370378-bib-0055] Khoshnood, S. , N. Sadeghifard , N. Mahdian , et al. 2023. “Antimicrobial Resistance and Biofilm Formation Capacity Among *Acinetobacter baumannii* Strains Isolated From Patients With Burns and Ventilator‐Associated Pneumonia.” Journal of Clinical Laboratory Analysis 37: e24814. 36573013 10.1002/jcla.24814 PMC9833984

[mbo370378-bib-0056] Kubin, C. J. , C. Garzia , and A. Uhlemann . 2025. “ *Acinetobacter baumannii* Treatment Strategies: A Review of Therapeutic Challenges and Considerations.” Antimicrobial Agents and Chemotherapy 69: e0106324. 40631987 10.1128/aac.01063-24 PMC12326985

[mbo370378-bib-0057] Kumar, M. , M. Agarwal , A. N. V. Lakshmi Kavya , et al. 2025. “Targeting MDR *Acinetobacter baumannii* With Phyto‐Synthesized Silver Nanoconjugates of *Valeriana wallichii*: A Mechanistic, In‐Silico, and Cytotoxic Investigation.” Microbial Pathogenesis 207: 107828. 40562283 10.1016/j.micpath.2025.107828

[mbo370378-bib-0058] Kumkoon, T. , M. Srisaisap , and P. Boonserm . 2023. “Biosynthesized Silver Nanoparticles Using *Morus alba* (White Mulberry) Leaf Extract as Potential Antibacterial and Anticancer Agents.” Molecules 28: 1213. 36770881 10.3390/molecules28031213 PMC9920803

[mbo370378-bib-0059] Lediga, M. E. , T. S. Malatjie , D. K. Olivier , D. T. Ndinteh , and S. F. Van Vuuren . 2018. “Biosynthesis and Characterisation of Antimicrobial Silver Nanoparticles From a Selection of Fever‐Reducing Medicinal Plants of South Africa.” South African Journal of Botany 119: 172–180.

[mbo370378-bib-0060] Liao, C. , Y. Li , and S. C. Tjong . 2019. “Bactericidal and Cytotoxic Properties of Silver Nanoparticles.” International Journal of Molecular Sciences 20: 449. 30669621 10.3390/ijms20020449 PMC6359645

[mbo370378-bib-0061] Mea, H. J. , P. V. C. Yong , and E. H. Wong . 2021. “An Overview of *Acinetobacter baumannii* Pathogenesis: Motility, Adherence and Biofilm Formation.” Microbiological Research 247: 126722. 33618061 10.1016/j.micres.2021.126722

[mbo370378-bib-0094] Meskini, M. , N. N. Goodarzi , S. Fereshteh , N. Bolourchi , A. Mirzaie , and F. Badmasti . 2022. “A Bioinformatics Approach to Introduce Novel Multi‐Epitope Vaccines Against *Acinetobacter baumannii* Retrieved from Immunogenic Extracellular Loops of Outer Membrane Proteins.” Informatics in Medicine Unlocked 31: 100989.

[mbo370378-bib-0062] Mickymaray, S. 2019. “One‐Step Synthesis of Silver Nanoparticles Using Saudi Arabian Desert Seasonal Plant *Sisymbrium irio* and Antibacterial Activity Against Multidrug‐Resistant Bacterial Strains.” Biomolecules 9: 662. 31661912 10.3390/biom9110662 PMC6920946

[mbo370378-bib-0063] Mishra, M. P. , and R. N. Padhy . 2018. “Antibacterial Activity of Green Silver Nanoparticles Synthesized From *Anogeissus acuminata* Against Multidrug Resistant Urinary Tract Infecting Bacteria In Vitro and Host‐Toxicity Testing.” Journal of Applied Biomedicine 16: 120–125.

[mbo370378-bib-0064] Moghadam, M. T. , S. Shahkolahi , I. A. Hashim , et al. 2025. “Engineered Phages and Engineered and Recombinant Endolysins Against Carbapenem‐Resistant Gram‐Negative Bacteria: A Focused Review on Novel Antibacterial Strategies.” Journal of Basic Microbiology 65: e70078. 40696543 10.1002/jobm.70078

[mbo370378-bib-0065] Moorthy, K. , K.‐C. Chang , P.‐J. Yu , et al. 2022. “Synergistic Actions of Phytonutrient Capped Nanosilver as a Novel Broad‐Spectrum Antimicrobial Agent: Unveiling the Antibacterial Effectiveness and Bactericidal Mechanism.” New Journal of Chemistry 46: 15301–15312.

[mbo370378-bib-0066] Moorthy, K. , K.‐C. Chang , W.‐J. Wu , J.‐Y. Hsu , P.‐J. Yu , and C.‐K. Chiang . 2021. “Systematic Evaluation of Antioxidant Efficiency and Antibacterial Mechanism of Bitter Gourd Extract Stabilized Silver Nanoparticles.” Nanomaterials 11: 2278. 34578594 10.3390/nano11092278 PMC8467971

[mbo370378-bib-0067] Moradi, H. , M. Ghavam , and A. Ghanbari . 2025. “Biosynthesis of Silver Nanoparticles Using *Thymus daenensis* Celak Against Wound Causing Microbes.” Waste and Biomass Valorization 16: 369–381.

[mbo370378-bib-0068] Mustapha, T. , and N. Misni . 2022. “A Review on Plants and Microorganisms Mediated Synthesis of Silver Nanoparticles.” Role of Plants Metabolites and Applications 19: 674. 10.3390/ijerph19020674 PMC8775445 35055505

[mbo370378-bib-0069] Naz, M. , A. Haider , M. Ikram , M. Z. Qureshi , and S. Ali . 2017. “Green Synthesis (*A. indica* Seed Extract) of Silver Nanoparticles (Ag‐NPs), Characterization, Their Catalytic and Bactericidal Action Potential.” Nanoscience and Nanotechnology Letters 9: 1649–1655.

[mbo370378-bib-0070] Nguyen, M. , and S. G. Joshi . 2021. “Carbapenem Resistance in *Acinetobacter baumannii*, and Their Importance in Hospital‐Acquired Infections: A Scientific Review.” Journal of Applied Microbiology 131: 2715–2738. 33971055 10.1111/jam.15130

[mbo370378-bib-0071] Ni, Q. , T. Zhu , W. Wang , et al. 2024. “Green Synthesis of Narrow‐Size Silver Nanoparticles Using *Ginkgo biloba* Leaves: Condition Optimization, Characterization, and Antibacterial and Cytotoxic Activities.” International Journal of Molecular Sciences 25: 1913. 38339192 10.3390/ijms25031913 PMC10856183

[mbo370378-bib-0072] Ontong, J. C. , S. Paosen , S. Shankar , and S. P. Voravuthikunchai . 2019. “Eco‐Friendly Synthesis of Silver Nanoparticles Using *Senna alata* Bark Extract and Its Antimicrobial Mechanism Through Enhancement of Bacterial Membrane Degradation.” Journal of Microbiological Methods 165: 105692. 31437555 10.1016/j.mimet.2019.105692

[mbo370378-bib-0073] Page, M. J. , J. E. McKenzie , P. M. Bossuyt , et al. 2021. “The PRISMA 2020 Statement: An Updated Guideline for Reporting Systematic Reviews.” BMJ (Clinical Research ed.) 372: n71. 10.1136/bmj.n71 PMC8005924 33782057

[mbo370378-bib-0074] Paosen, S. , S. Jindapol , R. Soontarach , and S. P. Voravuthikunchai . 2019. “ *Eucalyptus citriodora* Leaf Extract‐Mediated Biosynthesis of Silver Nanoparticles: Broad Antimicrobial Spectrum and Mechanisms of Action Against Hospital‐Acquired Pathogens.” APMIS 127: 764–778. 31512767 10.1111/apm.12993

[mbo370378-bib-0075] Rajangam, S. L. , and M. K. Narasimhan . 2024. “Current Treatment Strategies for Targeting Virulence Factors and Biofilm Formation in *Acinetobacter baumannii* .” Future Microbiology 19: 941–961. 38683166 10.2217/fmb-2023-0263 PMC11290764

[mbo370378-bib-0076] Ramya, E. , L. Jyothi , P. Vivek Vardhan , N. Sri Ram Gopal , and N. R. Desai . 2020. “Optical and Biomedical Applications of Eco‐Friendly Biosynthesized Silver Nano Spheres Using *Zingiber officinale* Root Extract.” Nano Express 1: 010021.

[mbo370378-bib-0077] Reyam, F. S. , and M. G. Ayad . 2021. “Biosynthesis and Characterization of Silver Nanoparticles Using *Cinnamomum zeylanicum* Extract and a Study of Antibacterial Effect Against Multi‐Drug Resistance Gram‐Negative Bacteria.” Biomedicine [publiée pour l'A.A.I.C.I.G.] 41: 249–255.

[mbo370378-bib-0078] Rodrigues, A. S. , J. G. S. Batista , M. Á. V. Rodrigues , et al. 2024. “Advances in Silver Nanoparticles: A Comprehensive Review on Their Potential as Antimicrobial Agents and Their Mechanisms of Action Elucidated by Proteomics.” Frontiers in Microbiology 15: 1440065. 39149204 10.3389/fmicb.2024.1440065 PMC11325591

[mbo370378-bib-0079] Sabzi, S. , M. Habibi , F. Badmasti , S. Shahbazi , M. R. Asadi Karam , and M. Farokhi . 2024. “Polydopamine‐Based Nano Adjuvant as a Promising Vaccine Carrier Induces Significant Immune Responses Against *Acinetobacter baumannii*‐Associated Pneumonia.” International Journal of Pharmaceutics 654: 123961. 38432452 10.1016/j.ijpharm.2024.123961

[mbo370378-bib-0080] Said, A. , M. Abu‐Elghait , H. M. Atta , and S. S. Salem . 2024. “Antibacterial Activity of Green Synthesized Silver Nanoparticles Using *Lawsonia inermis* Against Common Pathogens From Urinary Tract Infection.” Applied Biochemistry and Biotechnology 196: 85–98. 37099124 10.1007/s12010-023-04482-1 PMC10794286

[mbo370378-bib-0081] Salehi, S. , S. A. S. Shandiz , F. Ghanbar , et al. 2016. “Phytosynthesis of Silver Nanoparticles Using *Artemisia marschalliana* Sprengel Aerial Part Extract and Assessment of Their Antioxidant, Anticancer, and Antibacterial Properties.” International Journal of Nanomedicine 11: 1835–1846. 27199558 10.2147/IJN.S99882 PMC4857832

[mbo370378-bib-0082] Sati, A. , T. N. Ranade , S. N. Mali , H. K. Ahmad Yasin , and A. Pratap . 2025. “Silver Nanoparticles (AgNPs): Comprehensive Insights into Bio/Synthesis, Key Influencing Factors, Multifaceted Applications, and Toxicity—A 2024 Update.” ACS Omega 10: 7549–7582. 40060826 10.1021/acsomega.4c11045 PMC11886731

[mbo370378-bib-0083] Selvam, K. , A. Ragu Prasath , and A. K. Alanazi . 2026. “A Review of Recent Developments in Green Synthesis of Silver Nanoparticles: Antioxidant and Antibacterial Applications.” Microscopy Research and Technique 89: 134–147. 40815843 10.1002/jemt.70060

[mbo370378-bib-0084] Shamsoddini, S. M. , M. Davoudi , S. Shahbazi , and S. Z. Karizi . 2025. “Green Synthesis of Silver Nanoparticles Using *Acroptilon repens* Aqueous Extract and Their Antibacterial Efficacy Against Multidrug‐Resistant *Acinetobacter baumannii* .” Molecular Biology Reports 52: 47. 10.1007/s11033-024-10156-w 39666200

[mbo370378-bib-0085] Shirzadi‐Ahodashti, M. , Z. M. Mizwari , Z. Hashemi , et al. 2021. “Discovery of High Antibacterial and Catalytic Activities of Biosynthesized Silver Nanoparticles Using *C. fruticosus* (CF‐AgNPs) Against Multi‐Drug Resistant Clinical Strains and Hazardous Pollutants.” Environmental Technology & Innovation 23: 101607.

[mbo370378-bib-0086] Silva‐Holguín, P. N. , J. A. Garibay‐Alvarado , and S. Y. Reyes‐López . 2024. “Silver Nanoparticles: Multifunctional Tool in Environmental Water Remediation.” Materials (Basel, Switzerland) 17: 1939. 38730746 10.3390/ma17091939 PMC11084846

[mbo370378-bib-0087] Sterne, J. A. , M. A. Hernán , B. C. Reeves , et al. 2016. “ROBINS‐I: A Tool for Assessing Risk of Bias in Non‐Randomised Studies of Interventions.” BMJ 355: i4919. 27733354 10.1136/bmj.i4919 PMC5062054

[mbo370378-bib-0088] Thomas, S. , R. A. Gonsalves , J. Jose , S. H. Zyoud , A. R. Prasad , and J. Garvasis . 2024. “Plant‐Based Synthesis, Characterization Approaches, Applications and Toxicity of Silver Nanoparticles: A Comprehensive Review.” Journal of Biotechnology 394: 135–149. 39159752 10.1016/j.jbiotec.2024.08.009

[mbo370378-bib-0089] Wintachai, P. , S. Paosen , C. T. Yupanqui , and S. P. Voravuthikunchai . 2019. “Silver Nanoparticles Synthesized With *Eucalyptus citriodora* Ethanol Leaf Extract Stimulate Antibacterial Activity Against Clinically Multidrug‐Resistant *Acinetobacter baumannii* Isolated From Pneumonia Patients.” Microbial Pathogenesis 126: 245–257. 30445131 10.1016/j.micpath.2018.11.018

[mbo370378-bib-0090] Younas, M. , M. H. Rasool , M. Khurshid , et al. 2023. “ *Moringa oleifera* Leaf Extract Mediated Green Synthesis of Silver Nanoparticles and Their Antibacterial Effect Against Selected Gram‐Negative Strains.” Biochemical Systematics and Ecology 107: 104605.

[mbo370378-bib-0091] Zehra, S. H. , K. Ramzan , J. Viskelis , P. Viskelis , and A. Balciunaitiene . 2025. “Advancements in Green Synthesis of Silver‐Based Nanoparticles: Antimicrobial and Antifungal Properties in Various Films.” Nanomaterials (Basel, Switzerland) 15: 252. 39997815 10.3390/nano15040252 PMC11858222

[mbo370378-bib-0092] Zhang, S. , L. Di , Y. Qi , X. Qian , and S. Wang . 2024. “Treatment of Infections Caused by Carbapenem‐Resistant *Acinetobacter baumannii* .” Frontiers in Cellular and Infection Microbiology 14: 1395260. 39081869 10.3389/fcimb.2024.1395260 PMC11287075

