# Supplementary material for: Green‐Synthesized Silver Nanoparticles Against Acinetobacter baumannii: A Systematic Review of Antibacterial Activity, Mechanisms of Action, Toxicity, and Antibiofilm Effects
Source: Microbiologyopen. 2026 Aug 19;15(4):e70378. doi: 10.1002/mbo3.70378 (PMC13487469; doi:10.1002/mbo3.70378)
Supplement: Supplementary file 1 [file MBO3-15-e70378-s001.docx]

| **Table S2.** Risk of bias assessment of studies using ROBINS-I risk of bias tool (M: Moderate, L: Low, H: High). | | | | | | | | | |
| --- | --- | --- | --- | --- | --- | --- | --- | --- | --- |
|  |  |  |  |  |  |  |  |  |  |
| **Study Code** | **First authors** | **Bias due to confounding** | **Bias in classification of interventions** | **Bias in selection of participants** | **Bias due to deviations from intended interventions** | **Bias due to missing data** | **Bias in measurement of outcomes** | **Bias in selection of reported results** | **Overall Risk of Bias** |
| S1 | Abdellatif | M | L | L | M | M | M | M | M |
| S2 | Abdussalam-Mohammed | M | L | L | L | L | M | M | M |
| S3 | Abootalebi | M | L | L | M | L | M | M | M |
| S4 | Ajlouni | M | M | L | L | L | M | M | M |
| S5 | Akar | L | M | L | L | L | M | M | M |
| S6 | Akay | M | L | L | L | L | M | M | M |
| S7 | Akther | M | L | L | L | L | L | M | M |
| S8 | Alhamdi | M | L | L | L | L | M | M | M |
| S9 | Alharbi-1 | M | L | L | L | L | M | M | M |
| S10 | Alharbi-2 | M | L | L | L | L | M | L | M |
| S11 | Almudhafar | M | L | L | L | L | M | M | M |
| S12 | Al-Otibi | M | M | L | L | L | M | M | M |
| S13 | AlSalhi-1 | M | L | L | M | L | M | M | M |
| S14 | AlSalhi-2 | M | L | L | L | L | M | M | M |
| S15 | Alsharari | M | L | L | L | L | M | M | M |
| S16 | AL-Zubaidi | M | L | L | L | L | M | M | M |
| S17 | Aman | M | L | M | L | M | M | M | M |
| S18 | Arshad | M | L | L | L | L | M | L | M |
| S19 | Baghayeri | M | L | L | L | M | M | M | M |
| S20 | Barabadi | M | L | L | L | L | L | M | M |
| S21 | Behdad | M | L | L | M | L | M | M | M |
| S22 | Buapoon | M | M | L | L | L | M | M | M |
| S23 | Calhan | M | L | M | L | L | M | M | M |
| S24 | Ceylan | M | L | L | L | L | L | M | M |
| S25 | Choi | M | L | L | L | L | M | M | M |
| S26 | Deonas | M | M | L | M | L | M | L | M |
| S27 | Devanesan | M | L | L | L | L | M | M | M |
| S28 | Ebrahimzadeh | M | L | L | L | L | M | M | M |
| S29 | Eroglu | M | L | L | M | L | L | M | M |
| S30 | Gautam | M | L | L | M | L | M | M | M |
| S31 | Geyesa | M | L | L | L | L | M | M | M |
| S32 | Ghosh | M | L | L | L | L | M | M | M |
| S33 | Hashemi-1 | M | L | M | L | L | M | M | M |
| S34 | Hashemi-2 | M | L | M | L | L | M | M | M |
| S35 | Hashemi-3 | M | L | L | L | L | M | M | M |
| S36 | Hou | M | L | M | L | M | L | M | M |
| S37 | Hudaya-1 | M | L | L | L | L | M | M | M |
| S38 | Hudaya-2 | M | L | L | L | L | M | M | M |
| S39 | Khatami | M | L | L | L | L | L | L | M |
| S40 | Khojasteh‑Taheri | M | L | L | L | L | M | M | M |
| S41 | Kumar | M | L | M | L | L | M | M | M |
| S42 | Kumkoon | M | L | L | M | L | M | M | M |
| S43 | Lediga | M | L | L | L | L | M | M | M |
| S44 | Megdar-1 | M | L | M | L | L | M | M | M |
| S45 | Megdar-2 | M | L | M | L | L | M | M | M |
| S46 | Mickymaray | M | L | L | L | L | M | M | M |
| S47 | Mishra | L | L | M | L | L | M | L | M |
| S48 | Moorthy-1 | M | L | L | L | L | M | M | M |
| S49 | Moorthy-2 | M | L | L | L | L | M | M | M |
| S50 | Moradi | M | L | L | M | L | M | M | M |
| S51 | Naz | M | M | L | L | L | M | M | M |
| S52 | Ni | M | L | L | L | M | L | M | M |
| S53 | Nithin | M | L | M | L | L | M | M | M |
| S54 | Ontong | M | L | L | L | L | M | M | M |
| S55 | Paosen | L | L | M | L | L | M | L | M |
| S56 | Ramya | M | L | L | L | L | M | M | M |
| S57 | Said | M | L | L | M | L | L | M | M |
| S58 | Saleh | M | L | M | M | M | M | M | M |
| S59 | Salehi | M | L | L | L | L | M | M | M |
| S60 | Shamsoddini | M | L | M | L | L | M | M | M |
| S61 | Shirzadi-Ahodashti | M | L | L | L | L | M | L | M |
| S62 | Wintachai | M | L | L | M | L | M | M | M |
| S63 | Younas | M | L | M | L | L | M | L | M |
